# Spirohydantoin derivatives exert dopamine D_2_-like receptor-independent cytotoxicity in glioblastoma cells with possible involvement of calpain inhibition

**DOI:** 10.1038/s41598-026-43014-9

**Published:** 2026-03-24

**Authors:** Katarzyna Kucwaj-Brysz, Sabina Podlewska, Klaudia Jakubowska, Aleksandra Mąsior, Michał Wilczkowski, Beata Duszyńska, Justyna Drukała, Jadwiga Handzlik, Danuta Jantas

**Affiliations:** 1 Chair of Chemical Technology and Biotechnology of Drugs, Jagiellonian University Medical College, Medyczna 9, 30-688 Cracow, Poland; 2 Maj Institute of Pharmacology, Polish Academy of Sciences, Smetna 12, 31-343 Cracow, Poland; 3 Department of Cell Biology, Faculty of Biochemistry, Biophysics and Biotechnology, Jagiellonian University, Gronostajowa 7, 30-387 Cracow, Poland

**Keywords:** Glioblastoma multiforme, Dopamine receptors’ ligands, Calpain inhibitors, U87MG, A172, U138MG, Cancer, Drug discovery, Oncology

## Abstract

Glioblastoma multiforme (GBM) maintains one of the most aggressive brain tumor with increasing resistance. Herein in vitro study investigates spirohydantoin derivatives with diversified dopamine D_2_R-like affinity as potential anti-glioblastoma agents. Cytotoxic studies with three human GBM cell lines (U87MG, A172 and U138MG) and human skin fibroblasts as non-tumor control, led to choose compounds **4**, **6** and **7** with selective cell-damaging activity much greater than these observed for clinically used chemotherapeutic temozolomide (TMZ). The IC_50_ values for A172 cells were 56, 5, 21 and 513 µM for **4**, **6**, **7** and TMZ, respectively. Moreover, we demonstrated the potency of **4**, **6** and **7** as adjuvant therapeutics, even in GBM resistant phenotype. Interestingly, we observed a cell-damaging effect of calpain inhibitors in GBM cells and their higher cytotoxicity when combined with **4**, **6** and **7**, which together with molecular docking studies suggest that tested hydantoins might be also calpain inhibitors. Comparing the IC_50_ values between used GBM cell lines, dopamine receptors’ affinities and associating these data with expression level of D_2_-like receptors, we rather exclude the involvement of these receptors in anticancer mechanism of investigated compounds. Collectively, these results justify further investigation of compounds from hydantoin family as anti-glioblastoma candidates.

**Supplementary Information:**

The online version contains supplementary material available at 10.1038/s41598-026-43014-9.

## Introduction

Glioblastoma multiforme (GBM) is the most common and highly malignant brain tumour^1^. The median patient survival is in the range between 12 and 15 months, and the five-year survival rate remains below 10%, thus providing a very high mortality rate. It is estimated that annually there are 3–5 diagnosed cases per 100,000 individuals worldwide^2,3^. It is now 20 years after the establishment of the Stupp protocol for GBM treatment (surgical resection and radiotherapy in combination with chemotherapy using temozolomide (TMZ)), and no major clinical advances have been achieved; thus, increasing patients’ overall survival remains a great challenge^4^. Unfortunately, the clinical efficacy of TMZ, a DNA alkylating agent and still the gold standard for GBM treatment, is insufficient mainly due to two reasons: increasing GBM cell resistance to this drug^5^ and its limited ability to cross the blood-brain barrier (BBB)^6^. Therapy of recurrent GBM with bevacizumab, a monoclonal antibody targeting vascular endothelial growth factor receptor, despite its promising antiangiogenic profile, had a disappointing impact on overall survival^7,8^. Other than TMZ, known chemotherapies — carmustine and lomustine—are considered adjuvants and second-line therapy; however, these strategies increase median overall survival by only a few months, and more research is necessary^9–11^. Hence, GBM remains an incurable disease with minimal therapeutic advances and urgently needs searching for more effective, targeted therapy, including multimodal approaches.

**Fig. 1:**
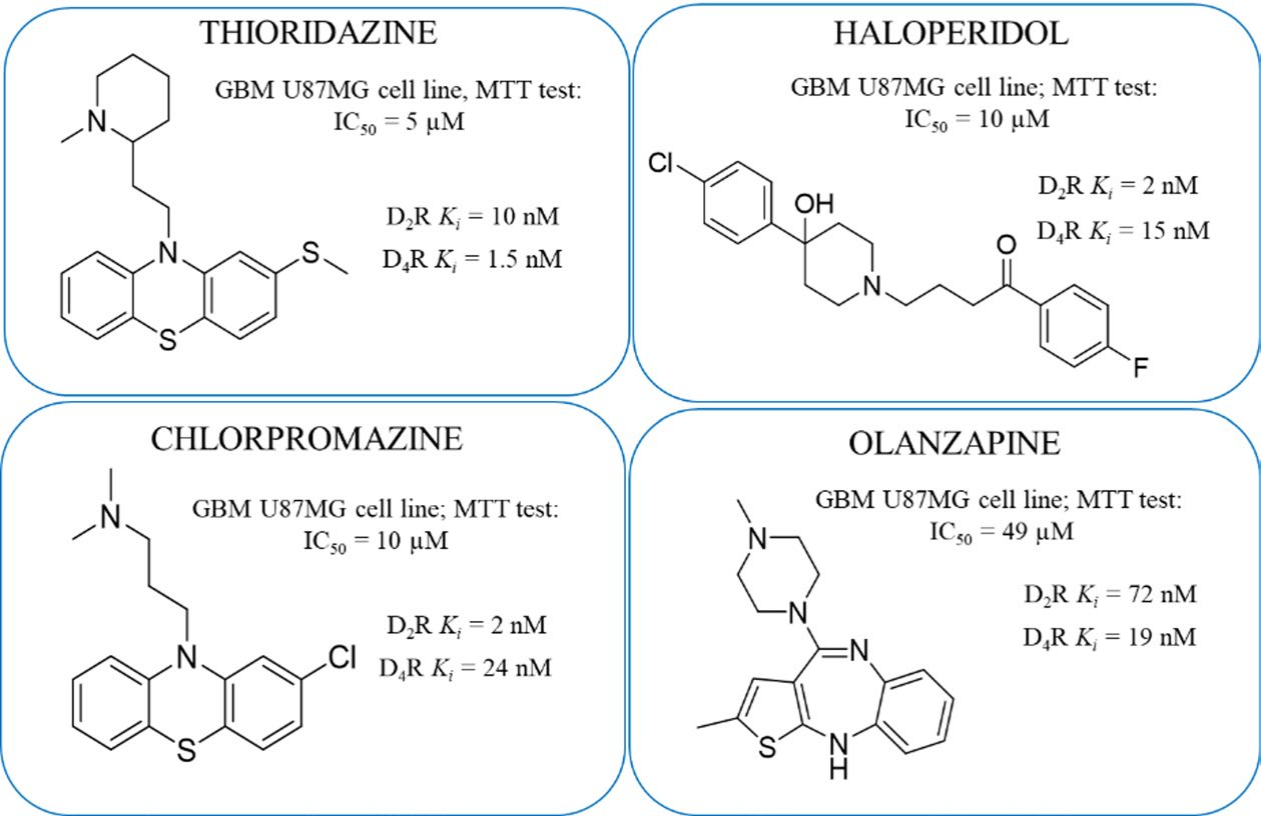
The dopamine D_2_ and D_4_ (D_2_R/D_4_R) receptors binding affinity and cytotoxic properties U87MG cells for representative antipsychotics (thioridazine, haloperidol, chlorpromazine, olanzapine)^19,30,31^.

GBM, like other tumours, is not an independent entity and depends strongly on its microenvironment. Signalling between tumour cells and the microenvironment drives GBM progress^12^. The brain, the location of GBM’s growth, is highly enriched in a neurochemical milieu (dopamine, serotonin, noradrenaline, acetylcholine, glutamate, gamma-aminobutyric acid (GABA), etc.), suggesting that their signalling pathways may profoundly impact tumour growth^13^. Excitedly, over the last few years, several lines of evidence have indicated the engagement of the dopaminergic system in the progression of this highly aggressive brain cancer^14^. The studies showed that the world-renowned antipsychotics, dopamine D_2_ receptor (D_2_R) antagonists, may be successfully repurposed as a new strategy to inhibit the growth of GBM cells (Fig. 1)^15–19^. Moreover, the impiridone anticancer agent ONC201, which has already entered clinical trials and is now being evaluated in Phase II in GBM patients, has recently been shown to act as a D_2_R/D_3_R antagonist^20–22^.

On the other hand, it is not clearly indicated whether the anti-glioblastoma activity of D_2_R antagonists is related to the modulation of D_2_R or rather *via* another protein target(s) of which ligands accidentally fit also to D_2_R ligands’ pharmacophore model^19^. For example, the compound ONC201, is a full antagonist of D_2_ and D_3_ receptors at micromolar concentrations which is similar to its anticancer activities^15,18^. Interestingly, other well-known D_2_R antagonists with high affinity to D_2_R (*K*_*i*_ at low nanomolar range) showed cytotoxic effect also at micromolar concentrations (Fig. 1). It is worth noting that indicated in Fig. 1 antipsychotics have also high, or even higher, affinity to D_4_ receptor (D_4_R) and in vivo studies using selective D_4_R ligands showed this receptor as promising target for glioblastoma therapy^23,24^. It should also be mentioned that there are also reports on possible therapeutic applications of D_3_R antagonists^25,26^ and D_1_R or D_5_R ligands for glioblastoma management^26–29^. However, these research areas are less recognized in comparison to the quite well-recognized role of D_2_R in this field.

**Fig. 2:**
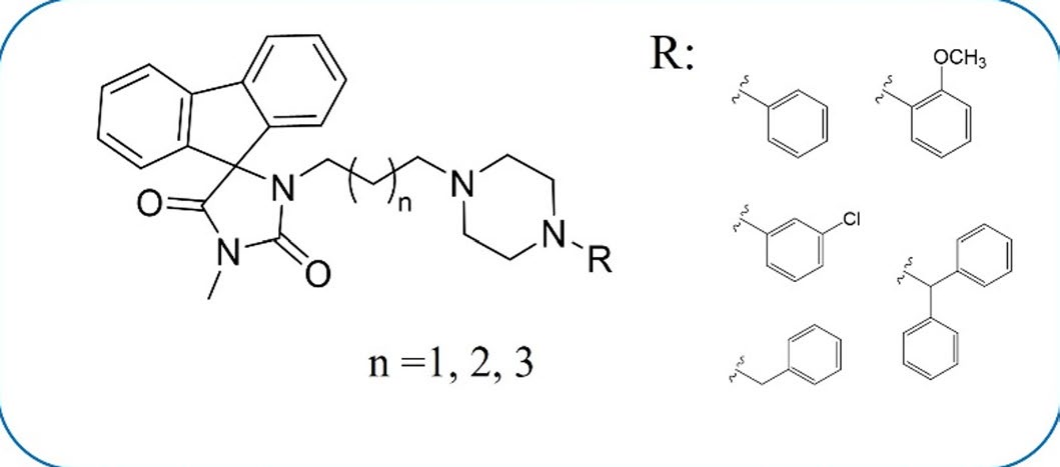
The general structure of the investigated 5-spirofluorenohydantoin derivatives, **1**–**8**. n – number of methylene group(s) in linker; R – aromatic substituent.

In the literature, there are no studies on whole libraries of D_2_-like receptors’ ligands. The tendency is to evaluate only the ligand(s) with the highest affinity for further biological studies^24,32,33^. Hence, it is not possible to analyse the correlation between D_2_-like receptors’ affinity and cytotoxic activity in GBM cells. For example, the less potent D_4_ ligands reported in Matteuci et al. studies^24,32^ may also have strong antitumour activity. The missing data are not enough to conclude that dopamine receptors’ affinity is really crucial for anti-GBM activity, or is it a coincidence, as the result of a similar pharmacophore that fits another protein target. Our study concerns the entire 8-membered hydantoin series from the in-house library, and their cytotoxic activity has been evaluated regardless of D_2_R affinity (13 nM < *K*_*i*_ < 973 nM) (Fig. 2) to address these two biological effects. Next, for the three compounds with the most significant cell-damaging effects, initial studies on the putative mechanism and potential synergism with TMZ have been conducted. Finally, molecular modelling studies were performed to identify potential anti-glioblastoma targets among the tested hydantoin derivatives, that may be hidden behind D_2_R affinity.

## Materials and methods

### Chemicals

The 5-spirofluorenohydantoin derivatives **1–8** (Fig. 2 and Table S1) were synthetized as described previously^34^ and delivered for biological testing by Chair of Chemical Technology and Biotechnology of Drugs, Jagiellonian University, Medical College (Cracow, Poland). High glucose Dulbecco’s Modified Eagle’s Medium (DMEM, #41966029), heat inactivated foetal bovine serum (FBS, #10500064), trypsin/EDTA (0.25% (#25200056), penicillin-streptomycin (#15140-122) and DPBS (Dulbecco’s phosphate-buffered saline, without calcium chloride and magnesium chloride, #14190144) were from Gibco (Life Technologies Ltd., Paisley, UK). The Cytotoxicity Detection Kit and Cell proliferation ELISA BrdU were from Roche Diagnostics GmbH (Mannheim, Germany). Caspase-3 (Ac-DEVD-AMC) fluorogenic substrate was obtained from Enzo Life Sciences (New York, NY, USA). TMZ (3-Methyl-4-oxoimidazo[5,1-d][1,2,3,5]tetrazine-8-carboxamide) and KU-55933 (2-(4-Morpholinyl)-6-(1-thianthrenyl)-4 H-pyran-4-one) were purchased from Apollo Scientific Ltd (Bredbury, Stockport, Cheshire, UK) and Adooq Bioscience (Irvine, CA, USA), respectively. BrdU Monoclonal Antibody (MoBU-1), Alexa Fluor^®^488 was purchased from Invitrogen (Life Technologies Ltd., Eugene, Oregon, USA). All other reagents were from Sigma (Sigma-Aldrich Chemie GmbH, Taufkirchen, Germany).

### Cell cultures

Human GBM cell lines: U87MG (ATCC HTB-14; RRID: CVCL_0022), A172 (ATCC CRL-1620; RRID: CVCL_0131), U138MG (grade IV, ATCC HTB-16; RRID: CVCL_0020) and human neuroblastoma SH-SY5Y cell line (ATCC CRL-2266; RRID: CVCL_0019) were purchased from American Type Culture Collection (Manassas, VA, USA). Frozen human skin fibroblasts (HSF) were from Prof. Justyna Drukała from the Department of Cell Biology, Faculty of Biochemistry, Biophysics and Biotechnology, Jagiellonian University (Cracow, Poland). HSF isolation and storage were done in accordance with the Jagiellonian University Bioethical Committee Agreement (1072.6120.9.2017). The HSF are accepted in the GBM field as a control cellular model of non-transformed cells to verify the specificity of observed cytotoxic effects of tested compounds^35–37^. The GBM cells, SH-SY5Y cells and HSF were cultured in T75 flasks in DMEM supplemented with 10% FBS, penicillin (100 IU/mL) and streptomycin (100 µg/mL) at 37 °C in an atmosphere with saturated humidity containing 95% air and 5% CO_2_. After reaching about 80% confluence, the cells were detached from the surface with 0.25% (GBM cells and HSF) or 0.05% (SH-SY5Y cells) trypsin/EDTA solution and further propagated in flasks or seeded into experimental plates. The GBM and HSF cells were manually counted with Bürker chamber and seeded at densities: 1 × 10^4^, 5 × 10^4^ and 5 × 10^5^ cells per well for 96-, 24- and 6-well plate format, respectively. After manual counting, the SH-SY5Y cells were seeded into a 6-well plates at a density of 1 × 10^6^ cells per well. The U87MG cells were used for experiments between passages 5–17, A172 and U138MG cells between passages 4- 8, HSF between passages 5–7 and SH-SY5Y cells between passages 4–6. The GBM and SH-SY5Y cells after rebanking were regularly tested for putative Mycoplasma contamination with MycoBlue™ Mycoplasma Detector (Vazyme Biotech Co. Ltd., Nanjing, China).

### Cell treatment

**Fig. 3:**
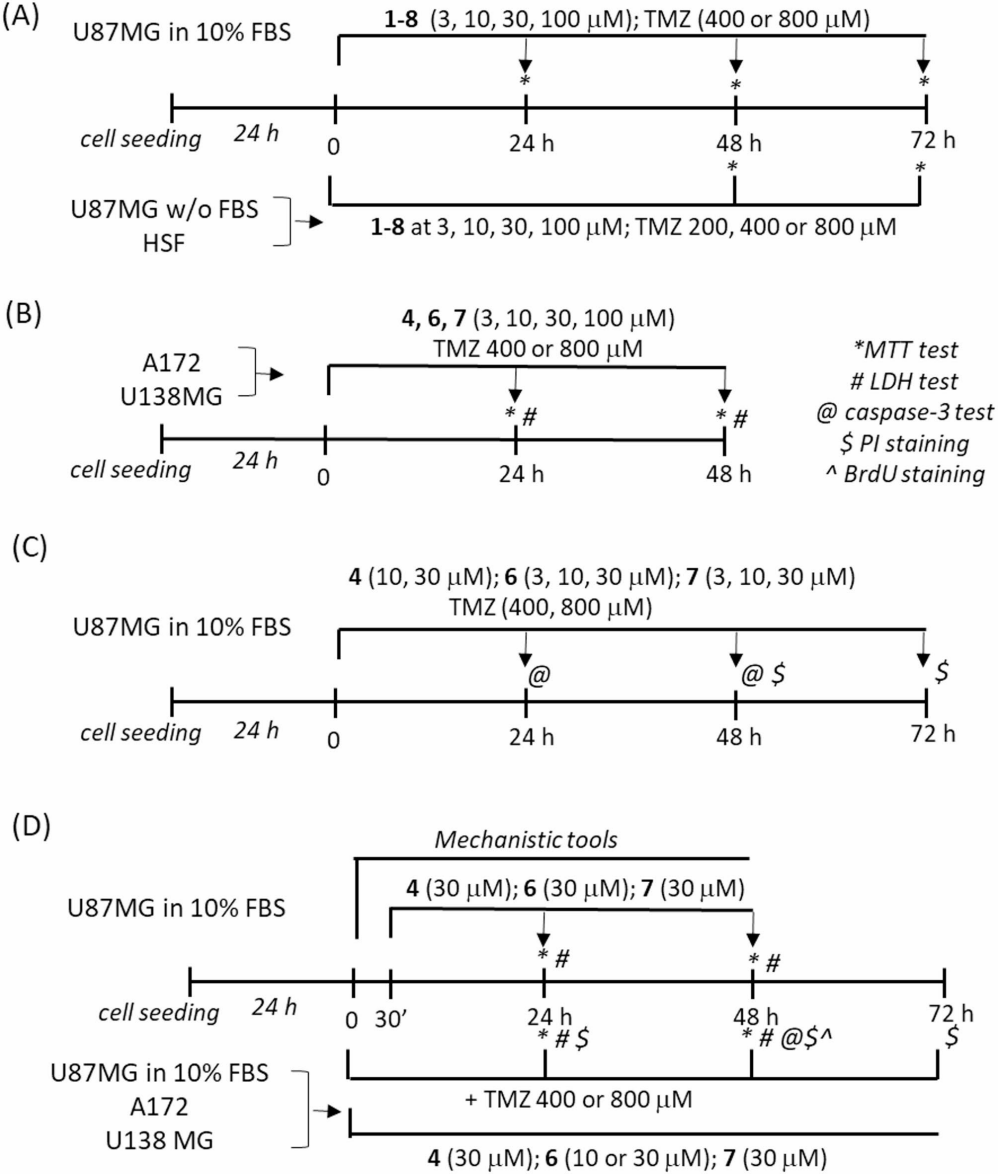
The graphical presentation of experimental design.

The experimental design is presented on Fig. 3.

One day after cell seeding into 96-well experimental plates (1 × 10^4^ cells/well), the U87MG cells growing in medium with 10% FBS as well with medium devoid of serum, and HSF were treated with compounds **1**–**8** as presented in Fig. 3A. For U87MG cells, we used a serum starvation model (medium w/o FBS) to distinguish between cytostatic and cytotoxic effects of the tested compounds. It has been shown that under such serum-starvation conditions, cells not only proliferate more slowly but also exhibit an increased basal level of autophagic flux compared with cells cultured in medium containing 10% FBS^38^.

In A172 and U138MG cells, we tested the effects of **6**, **4** or **7** as presented in Fig. 3B. In order to test impact of the best acting GBM-damaging compounds on apoptotic (caspase-3 activity) and necrotic (PI staining) and compare their effectiveness to TMZ, we performed experiments in U87MG cells as illustrated on Fig. 3C. For mechanistic studies the U87MG cells were pre-treated for 30 min with: an antioxidant - N-acetyl-cysteine (NAC, 1 mM); caspase-3 inhibitor - Ac-DEVD-CHO (20 µM); necroptosis inhibitor - Necrostatin-1 (Nec-1, 20 µM); ferroptosis inhibitor - Ferrostatin-1 (Fer-1, 20 µM); ATM inhibitor - KU55933 (KU, 1 µM), mTOR inhibitor - rapamycin (Rap, 0.1 µM); JNK inhibitor - SP600125 (JNKi, 10 µM); ERK1/2 inhibitor - PD98052 (PD, 20 µM); PI3-K/Akt inhibitor - LY294002 (LY, 10 µM); cathepsin D inhibitor - pepstatin A (0.3 µM) and calpains inhibitors: MDL28170 (MDL, 20 µM) and calpeptin (Calp, 20 µM) alone or in combination with 30 µM of **6**, **4** or **7** (Fig. 3D).

To test the effect of combined treatment of investigated compounds with TMZ, in separate experimental sets, the U87MG, A172 and U138MG cells were treated for 24–72 h with a combination of TMZ and **6**, **4** or **7** as pictured on Fig. 3D.

Before biological testing, the hydantoin-phenylpiperazine compounds **1–8** were first dissolved in DMSO to a concentration of 50 mM. Further diluted stocks (0.3, 1, 3 and 10 mM) of these compounds were tested in H_2_O, 1:1 H_2_O/DMSO mixture or DMSO to reduce a content of DMSO in solvent since it could significantly impede in vitro and in vivo pharmacological evaluation of new drugs and is not optimal for their possible further clinical development^39,40^. None of the **1–8** compounds from 50 mM stocks were soluble in H_2_O; the **1**, **2**, **4**, **6**, and **7** stock solutions were prepared in a 1:1 H_2_O/DMSO mixture, and the **3**, **5**, and **8** stock solutions were insoluble in water and thus were finally prepared in DMSO. TMZ stock solution (100 mM) was prepared in DMSO, and subsequent dilutions were made in a 1:1 H_2_O/DMSO mixture. The **1–8** and TMZ stocks were stored at 4 °C. NAC stock solution (100 mM) was prepared in sterile distilled water and stored at -20 °C. Ac-DEVD-CHO (20 mM), Nec-1 (100 mM), Fer-1 (100 mM), Rap (100 mM), LY294002 (10 mM), pepstatin A (10 mM), MDL28170 (20 mM) and calpeptin (20 mM) stock solutions were prepared in DMSO and stored at -20 °C. They were further diluted in distilled water before experiments. PD98052 (10 mM), KU55933 (3 mM), and SP600125 (10 mM) stock solutions were prepared in DMSO and stored at -20 °C, and were further diluted before experiments in a 1:1 H_2_O/DMSO mixture. Each experimental set of the control cultures was supplemented with the appropriate vehicle (DMSO or 1:1 H_2_O/DMSO mixture), and the solvent was present in cultures at a final concentration of 1%. All agents were sterilized with a syringe filter (pore diameter 0.2 μm) and were added to the culture medium under light-limited conditions.

### The MTT reduction assay

The cell viability was assessed by 3-(4,5-dimethylthiazol-2-yl)-2,5-diphenyltetrazolium bromide (the MTT) assay as previously described^41^. This is a biochemical method for indirect assessment of cell viability and/or cell proliferation. Briefly, 10 µL of the MTT solution (1.5 mg/mL in DPBS) was added per well after a particular time of cell treatment in 96-well plates and incubated at 37 °C for 60 min (until blue formazan crystals were visible). After medium removal, 100 µL of DMSO was added to each well to solubilize the crystals and the absorbance of probes at 570 nm was measured in a microplate multi-well reader (Infinite^®^ M200 PRO, Tecan Austria GmbH, Grodig, Austria). The data were normalized to the vehicle-treated cells (100%) and are presented as the mean ± SEM from 4 to 5 independent experiments with 3–5 replicates.

### The LDH release assay

Cytotoxicity was assessed using the LDH release assay as described previously^41^. Briefly, after a particular time of cell treatment in 96-well plates, 50 µL of medium from each well was collected into a new 96-well plate and stored for up to 1 h at 4 °C. Twenty-five microliters of the LDH mixture was added to each well and incubated at room temperature for 10–15 min. The absorbance of probes at 490 nm was measured in a microplate multi-well reader (Infinite^®^ M200 PRO, Tecan Austria GmbH, Grodig, Austria). The data were normalized to the vehicle-treated cells (100%) and are presented as the mean ± SEM from 4 to 5 independent experiments with 3–5 replicates.

### PI staining and flow cytometry

The U87MG cells growing in the 24-well format, one day after cell seeding (5 × 10^4^ cells/well), were treated for 48 and 72 h with **6** (3, 10 and 30 µM), **4** (10 and 30 µM), **7** (3, 10 and 30 µM) and TMZ (800 µM). In separate experimental sets, the cells were treated for 48 h with a combination of TMZ (800 µM) and **6** (3, 10, and 30 µM), **4** (10 and 30 µM), or **7** (3, 10, and 30 µM). After cell treatment, they were stained with propidium iodide (PI), a marker of cells with damaged plasma membranes as described previously^41^. Briefly, the cells collected in 1.5 mL Eppendorf tubes were incubated with PI solution (1.5 µM) for 12 min at room temperature, centrifuged (for 3 min at 1300 rpm at 4°C), washed twice in ice-cold DPBS, and suspended in 200 µL of ice-cold DPBS. 1 × 10^4^ cells from each experimental group were analyzed with the BD FACSCanto II system and BD FACSDiva™ v5.0.1 software (BD Biosciences, San Jose, CA, USA) in the fluorescence channel for PerCP-Cy5-5-A (red fluorescence). Data are shown as a percentage of PI-positive cells (mean ± SEM) from 3 to 4 independent experiments with two replicates.

### Caspase-3 activity measurement

The U87MG cells were grown in the 6-well format (5 × 10^5^ cells/well) and were treated for 24 and 48 h with **6** (3, 10 and 30 µM), **4** (30 µM), **7** (10 and 30 µM) and TMZ (400 and 800 µM). In separate experimental sets, the cells were treated for 48 h with a combination of TMZ (400 or 800 µM) with **6** (10 and 30 µM), **4** (30 µM) or **7** (30 µM). The cells after medium removal and washing with ice-cold DPBS were stored at −20 °C. The cell lysates were prepared in ice-cold CAB (Caspase Assay Buffer) containing leupeptin (10 µg/mL) and pepstatin A (2 µg/mL). Caspase-3 activity was measured with fluorogenic substrate Ac-DEVD-AMC (50 µM) as described previously^41^. The fluorescence was measured in a multi-well microplate reader (Infinite^®^ M200 PRO, Tecan Austria GmbH, Grodig, Austria) with excitation and emission wavelengths of 360 nm and 460 nm, respectively. The data (expressed as the mean relative fluorescence units, RFU) were first normalized to the protein level (measured by the BCA method) and further calculated as a percentage of vehicle-treated cells and presented as the mean ± SEM from 3 to 4 separate experiments with two replicates.

### Proliferation assay

The U87MG cells growing in the 24-well format (5 × 10^4^ cells/well), one day after cell seeding, were treated for 48 h with **6** (3, 10 and 30 µM), **4** (10 and 30 µM) or **7** (10 and 30 µM) alone or in combination with TMZ (400 µM). After cell treatment, BrdU (Bromo-2′-deoxyuridine, 10 µM) was added to each well for 2 h. Next, the cells were washed, fixed (70% ethanol at − 20 °C for 24 h), denatured and immunostained with BrdU-mouse monoclonal antibodies-clone MoBU-1 conjugated to Alexa Fluor^®^488 as described in detail previously^41^. The cells (1 × 10^4^) were analyzed using the BD FACSCanto II system and BD FACSDiva™ v5.0.1 software (BD Biosciences, San Jose, CA, USA) in the fluorescence channel for FITC (green fluorescence). The data are presented as a percentage of BrdU-positive cells (mean ± SEM) from 3 independent experiments with 2 replicates.

### Radioligand binding assays - evaluation of affinities to D_2_R, 5-HT_1A_R, 5-HT_2A_R, 5-HT_7_R

HEK293 cells stably expressing the human 5-HT_1A_, 5-HT_6_, 5-HT_7b_ and D_2_ receptors and CHO-K1 cells expressing 5-HT_2A_R (prepared with the use of the Lipofectamine 2000) were maintained at 37 °C in a humidified atmosphere with 5% CO_2_ and grown in DMEM containing 10% dialysed FBS and 500 µg/mL G418 sulphate. For membrane preparation, the cells were subcultured in 150 cm^2^ flasks, grown to 90% confluence, washed twice with phosphate-buffered saline (PBS) prewarmed to 37 °C and pelleted by centrifugation (200 g) in PBS containing 0.1 mM EDTA and 1 mM dithiothreitol. Before membrane preparation, the pellets were stored at − 80 °C. The cell pellets were thawed and homogenized in 10 volumes of assay buffer using an Ultra Turrax tissue homogenizer and centrifuged twice at 35,000 g for 20 min at 4 °C, with a 15 min incubation at 37 °C between spins. The composition of the assay buffer is as follows: 5-HT_1A_R – 50 mM Tris–HCl, 0.1 mM EDTA, 4 mM MgCl_2_, 10 µM pargyline and 0.1% ascorbate; 5-HT_2A_R – 50 mM Tris–HCl, 0.1 mM EDTA, 4 mM MgCl_2_, and 0.1% ascorbate; 5-HT_7B_R– 50 mM Tris–HCl, 4 mM MgCl_2_, 10 µM pargyline and 0.1% ascorbate, D_2_R − 50 mM Tris-HCl, 1 mM EDTA, 4 mM MgCl_2_, 120 mM NaCl, 5 mM KCl, 1.5 mM CaCl_2_ and 0.1% ascorbate. The assays were incubated in a total volume of 200 µL on 96-well microtiter plates for 1 h at 37 °C, except for 5-HT_1A_R and 5-HT_2A _R,  which were incubated at room temperature). The equilibration process was terminated by rapid filtration through Unifilter GF/B plates (96-well cell harvester, PerkinElmer), and the radioactivity retained on the filters was quantified using a Microbeta plate reader (PerkinElmer). For displacement studies, the assay samples contained the following as radioligands: 2.5 nM [^3^H]-8-OHDPAT (187 Ci/mmol; 6919 GBq/mmol) – 5-HT_1A_R; 1 nM [^3^H]-ketanserin (53.4 Ci/mmol; 1975.8 GBq/mmol) − 5-HT_2A_R; 0.8 nM [^3^H]-5-CT (39.2 Ci/mmol; 1450.4 GBq/mmol) − 5-HT_7B_R; [^3^H]-raclopride (74.4 Ci/mmol) − D_2_R. Nonspecific binding was defined with 10 µM of 5-HT (5-HT_1A_R, 5-HT_7B_R). 10 µM of chlorpromazine (5-HT_2A_R), or 1 mM of (+)-butaclamol (D_2_R). Each compound was tested at 7 concentrations (10^–10^-10^− 4^ M). The inhibition constants (*K*_*i*_) were calculated from the *Cheng*-*Prusoff* equation^42^.

### Evaluation of specific binding to D_4_R

The D_4_ receptor binding was determined by Eurofins Discovery (https://emea.eurofinsdiscovery.com/) as a commercial service according to the internal SOP described below^43^.

Cell membrane homogenates (80 µg protein) are incubated for 60 min at 22 °C with 0.3 nM [^3^H]methyl-spiperone in the absence or presence of the test compound in a buffer containing 50 mM Tris-HCl (pH 7.4), 120 mM NaCl, 5 mM KCl, 5 mM MgCl_2_ and 1 mM EDTA. Nonspecific binding is determined in the presence of 10 µM (+)butaclamol. Following incubation, the samples are filtered rapidly under vacuum through glass fiber filters (GF/B, Packard) presoaked with 0.3% PEI and rinsed several times with ice-cold 50 mM Tris-HCl using a 96-sample cell harvester (Unifilter, Packard). The filters are dried, then counted for radioactivity in a scintillation counter (Topcount, Packard) using a scintillation cocktail (Microscint 0, Packard). The results are expressed as a percent inhibition of the control radioligand-specific binding. The standard reference compound is clozapine, which is tested in each experiment at several concentrations to obtain a competition curve from which its IC_50_ is calculated.

### Western blot

For measurement of D_2_-like receptors (D_2_R, D_3_R and D_4_R), the GBM cell lines (U87MG, A172 and U138 MG) and human neuroblastoma SH-SY5Y cells (used as a reference cell line with dopaminergic phenotype) were seeded into 6-well plate format at densities 5 × 10^5^ (GBM cells) and 1 × 10^6^ (SH-SY5Y cells) in cell culture medium containing 1% of antibiotics and 10% FBS. 48 h after seeding, the cells were washed twice with ice-cold DPBS and lysed (60 µL/well) with RIPA buffer supplemented with protease inhibitors cocktail (Roche). The protein level in cell lysates was measured using the Pierce^™^ BCA Protein Assay (Thermo Scientific, Rockford, USA) according to the manufacturer’s protocol. Western blot was performed on a ProteinSimple Jess, an automated capillary-based western blot platform (Bio-Techne, Minneapolis, MN, USA), according to the manufacturer’s instructions. A 12–230 kDa separation microplate kit (# SM-FL004, Bio-techne) was used with 1 µg/µL protein loaded onto the plate. Primary antibodies included rabbit anti-D_2_R antibody (1:50, sc-9113 Santa Cruz), rabbit anti-D_3_R antibody (1:50, ab42114 Abcam) and mouse anti-D_4_R antibody (clone 2B9) (1:50, MABN125 Sigma Aldrich). A chemiluminescence signal was detected using Anti-Rabbit (#DM-001, Bio-techne) and Anti-Mouse (#DM-002, Bio-techne) Detection Modules as described in the manufacturer’s manuals. Total protein was measured with the Protein Normalization Module (#DM-PN02, Bio-techne). Densitometric analysis of bands of interest was performed using the manufacturer-provided Compass software and normalized by the system to measure total protein levels. The data are expressed as the mean corrected area ± SEM from two independent experiments, each with triplicates.

### Statistical analysis

The data were analysed with TIBCO Statistica™ 14.0.0 (RRID: SCR_014213; TIBCO Software Inc., San Ramon, CA 94583, USA). Analysis of variance (one- or two-way ANOVA) and Duncan post hoc test for multiple comparisons were used to assess the statistical significance at *P* < 0.05. The IC_50_ values were calculated from results of the MTT test after 48 h of treatment for U87MG cells (cultured in medium with and w/o 10% FBS), human skin fibroblasts (HSF), A172 and U138MG cells using GraphPad Prism 5 (RRID: SCR_002798; GraphPad Software, Boston, MA 02110, USA) with chosen option Nonlineral regression log(inhibitor) vs. normalized response - Variable slope.

### Molecular modelling and BBB permeability calculations

Docking studies targeting µ-calpain were performed using its crystal structure of PDBID: 3BOW. Before docking, the structure underwent preparation using the Protein Preparation Workflow from the Schrödinger Suite (release 2024-4 The Induced Fit Docking protocol was applied in the extra precision mode. The compounds were prepared for docking using LigPrep from the Schrödinger Suite: protonation states were generated at pH 7.4 ± 0.0, and all possible stereoisomers were enumerated; other settings remained at default. Ligand-protein contacts were examined with the use of the Ligand-Protein Interaction panel. Visualization of the docking poses was conducted using PyMOL (version 2.5.2). The binding energies were estimated by combining molecular mechanics energies with implicit solvation models, applying the Molecular Mechanics – Generalized Born Surface Area (MM-GBSA) approach using Prime. The protocol was applied to the complexes, which were then subjected to molecular dynamics (MD)-based minimization. MD simulations of 200 ns were carried out in Desmond using the TIP3P solvent model and default settings. In addition, BBB permeability was assessed using QikProp.

## Results

*Cytotoxic effects of* ***1–8*** *spirohydantoin derivatives in GBM cell lines and in human skin fibroblasts*.

**Fig. 4:**
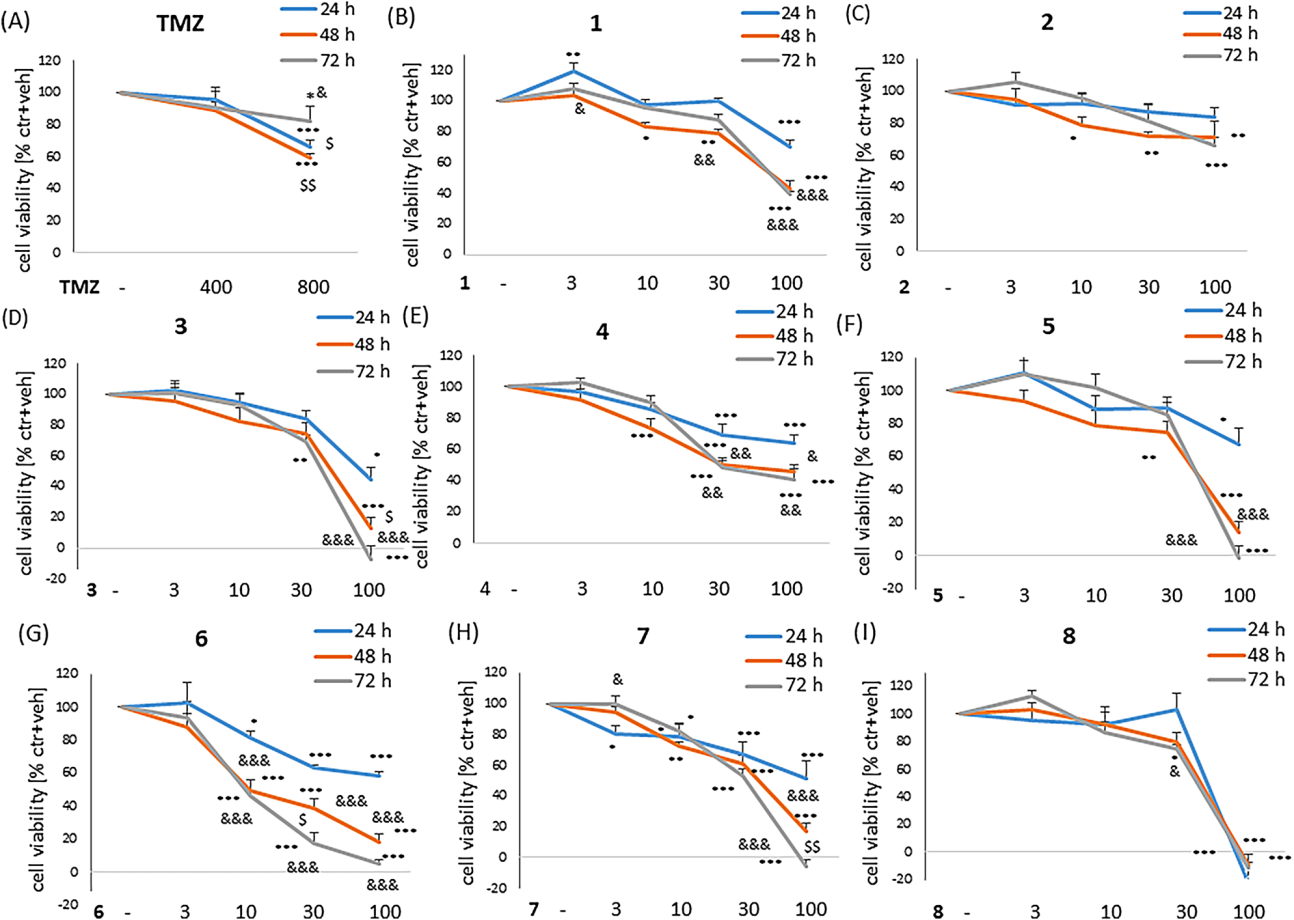
The cytotoxic effect of temozolomide (TMZ) and compounds **1–8** in U87MG cells cultured in medium with 10% FBS. The cells were treated with TMZ (400 and 800 µM) or particular **1–8** compounds (concentrations of 3, 10, 30 and 100 µM) for 24–72 h. Cell viability was assessed using the MTT reduction assay. The data were normalized to vehicle-treated cells and are presented as mean ± SEM from 5 independent experiments. ^*^P < 0.05, ^**^P < 0.01and ^***^P < 0.001 vs. vehicle-treated cells; ^&^*P* < 0.05, ^&&^*P* < 0.01 and ^&&&^*P* < 0.001 vs. 24 h; ^$^*P* < 0.05 and ^$$^*P* < 0.01and vs. 72 h.

First, the potential anticancer activities of **1–8** hydantoin-phenylpiperazine derivatives and TMZ were tested in U87MG cells cultured in medium containing 10% FBS (cell proliferation-favouring condition) (Fig. 4) and in medium devoid of FBS (cell proliferation inhibitory condition) (Fig. S1). In the former one condition (10% FBS), the TMZ evoked a significant cell damage of U87MG cells at a concentration of 800 µM but not 400 µM, and this effect was significantly lower after 72 h (about 20%) when compared to 24–48 h of treatment (about 40%) (Fig. 4A). Among tested hydantoin-phenylpiperazine compounds, we found a highest cell-damaging effects for **6**, **7** and **4** which were concentration- and time-dependent (Fig. 4G, H, E). A relatively middle cell-damaging effects was demonstrated by **1**, **3**, **5** and **8** (Fig. 4B, D, F, I) and the lowest **2** (Fig. 4C).

Under cell-proliferation-inhibitory conditions (medium w/o FBS), we observed that TMZ (400 µM) decreased cell viability by about 20% after 72 h of treatment in U87MG cells (Fig. S1A). Among tested hydantoin-phenylpiperazine compounds under this cell culture condition, we evidenced the highest concentration- and time-dependent cell-damaging effects for **6** and **7** (Fig. S1G, H). A relatively middle cell-damaging effect was demonstrated by compounds **1**, **3**, **4**, **5** and **8** (Fig. S1B, D, E, F, I), and the lowest **2** (Fig. S1C).

In another set of experiments, we investigated the potential cytotoxic effect of hydantoin-phenylpiperazine compounds **1–8** in HSF. In these cells, after 48 and 72 h of treatment, we did not observe any cytotoxic effect for TMZ (400 and 800 µM) and 3-100 µM of **1**, **2** and **4** (Fig. S2A, B, C, E). A low cell-damaging effect in HSF we found for compounds **6** and **7** (Fig. S2G, H) and moderate for **3**, **5** and **8** (Fig. S2D, F, I).

Next, for each compound, we determined IC_50_ values (based on MTT results after 48 h with GraphPad Prism 5) for U87MG and HSF cells and calculated the cancer index (CI) as the ratio of the IC_50_ in HSF cells to the IC_50_ in U87MG cells cultured with 10% FBS (Table 2). Based on IC_50_ values, CI and solubility of tested **1–8** compounds (preferred compounds soluble in H_2_O/DMSO mixture), the most promising GBM cell-damaging compounds appeared to be compounds **6**, **7** and **4** and they were more active than the reference clinical drug TMZ (Table 1).

As a follow-up investigation, we examined the effects of 24 and 48 h incubation of **6**, **7**, and **4** at a concentration range of 3-100 µM with two additional GBM cell lines: A172 and U138MG (IV grade). We found that TMZ (400 and 800 µM) reduced cell viability in a concentration- and time-dependent manner (Fig. S3A) and increased LDH release (Fig. S3B) in A172 cells. Among the three tested hydantoin-phenylpiperazine compounds in A172 cells, we observed the highest concentration- and time-dependent cell-damaging effect with **6**, as evidenced by MTT reduction (Fig. S3C) and LDH release assays (Fig. S3D). A relatively moderate cell-damaging effect was demonstrated by **7** (Fig. S3E, F), and the lowest by **4** (Fig. S3G, H). These observations are mirrored by the calculated IC_50_ values from the MTT assay after 48 h (Table 2), and A172 cells appeared to be more vulnerable to the cytotoxic action of **6**,** 7**, and TMZ compared to U87MG (Table 1).

**Table 1:** The IC_50_ values for inhibition of cell viability of U87MG cells and human skin fibroblasts (HSF) for hydantoin-phenylpiperazines compounds **1–8** and TMZ with calculated cancer index (CI).

Compound	Solvent	U87MG in 10% FBS	U87MG w/o FBS	HSF	CI [HSF vs.10% U87MG]
1	H_2_O/DMSO	81	111	> 1000	> 12
2	H_2_O/DMSO	808	107	> 1000	1.24
3	DMSO	46	65	60	1.3
**4**	H_2_O/DMSO	**52**	**97**	**> 1000**	**19.2**
5	DMSO	44	57	82	1.9
**6**	H_2_O/DMSO	**15**	**34**	**107**	**7.1**
**7**	H_2_O/DMSO	**33**	**88**	**243**	**7.4**
8	DMSO	43	38	51	1.2
TMZ	H_2_O/DMSO	831	> 1000	> 1000	1.2

CI, Cancer index; DMSO, Dimethyl sulfoxide; H_2_O/DMSO, 1:1 Mixture of distilled water and DMSO; TMZ, Temozolomide. The IC_50_ values [µM] were calculated from results of the MTT test after 48 h of treatment for U87MG cells (cultured in medium with and w/o 10% FBS) and in human skin fibroblasts (HSF) using Graph Pad Prism 5 (Nonlinear regression log(inhibitor) vs. normalized response - Variable slope). CI for each compound was calculated as the ratio of its IC_50_ in HSF cells to its IC_50_ in U87MG cells cultured with 10% FBS.

Studies performed in grade IV GBM, U138MG cells with TMZ (400 and 800 µM) showed that after 24 h of incubation, there is no induction of cell damage, and after 48 h we observed about 35% decrease in cell viability (Fig. S4A) but without an increase in the LDH level (Fig. S4B). Among the three tested hydantoin-phenylpiperazine compounds in U138MG cells, we observed the greatest concentration- and time-dependent cell-damaging effect for **6**, as evidenced by MTT reduction (Fig. S4C) and LDH release assays (Fig. S4D). A relatively moderate cell-damaging effects were demonstrated by **7** (Fig. S4E, F). In contrast, compound **4** at concentrations 3-100 µM did not evoke reduction in cell viability after 24 and 48 h of treatment (Fig. S4G). However, at a concentration of 100 µM, it evoked a significant increase in LDH levels after 48 h of treatment (Fig. S4G). The IC_50_ values calculated from the MTT assay after 48 h show that U138MG cells are less susceptible to the cell-damaging effects of **6**, **7**,** 4** and TMZ than A172 cells (Table 2) and U87MG cells (Table 1).

**Table 2:** The IC_50_ values for cell viability of compounds **4**, **6**,** 7**, and TMZ in A172 and U138MG cells.

Compound	A172	U138MG
**4**	56	> 1000
**6**	5	27
**7**	21	57
TMZ	513	825

The IC_50_ values [µM] were calculated from results of the MTT test after 48 h of treatment with compounds **4**, **6**,** 7**, and temozolomide (TMZ) in A172 and U138MG cells using Graph Pad Prism 5 (Nonlinear regression log(inhibitor) vs. normalized response - Variable slope.

### Study on mechanisms of GBM cell-damaging effects of compounds **6**, **7** and **4**

**Fig. 5:**
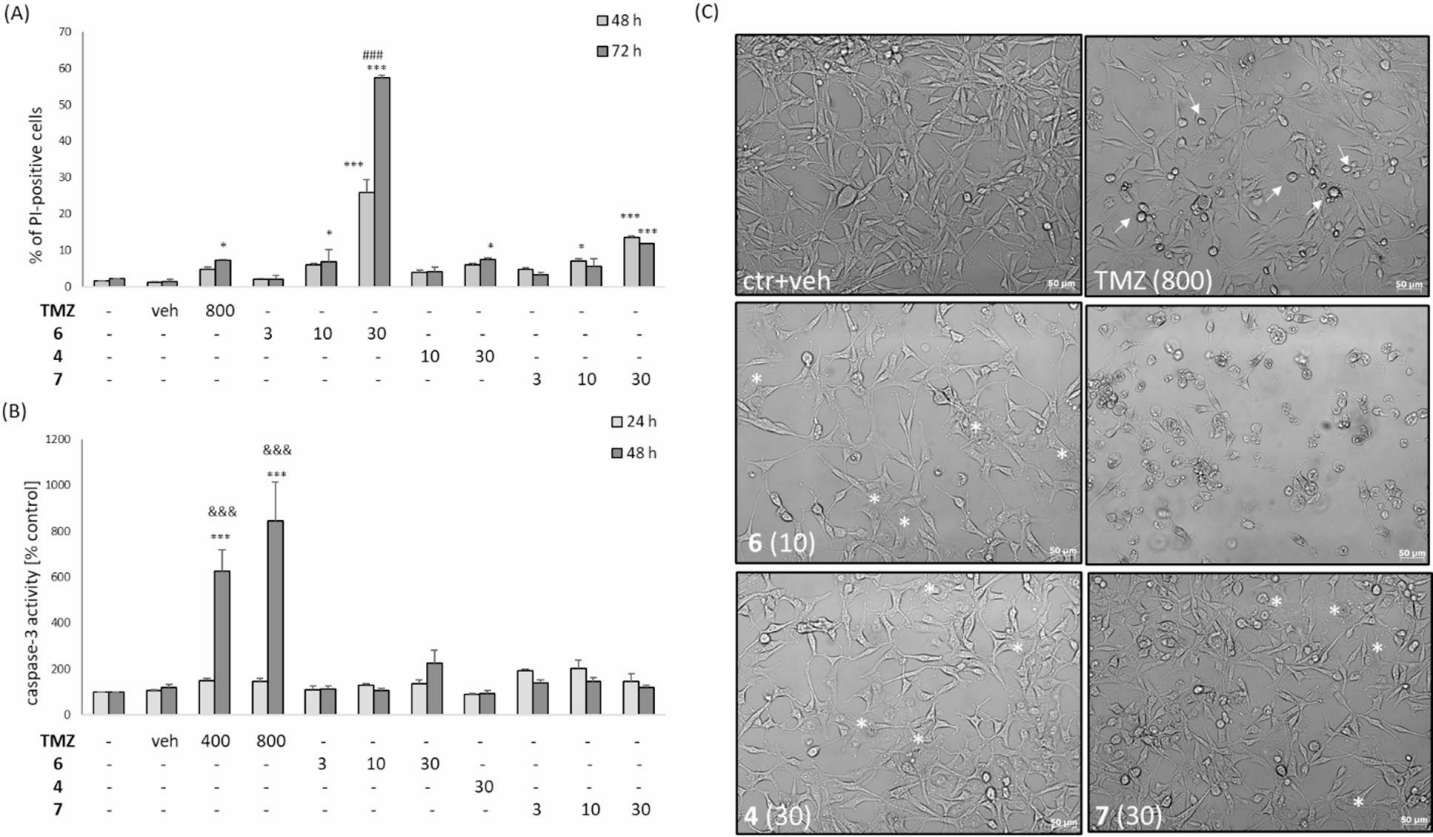
(**A**, **B**) The effects of compounds **6**, **4** and **7** on necrotic (**A**) and apoptotic (**B**) markers. The cells were treated with TMZ (400 and 800 µM) or compounds **6** (3–30 µM), **4** (30 µM) or **7** (3–30 µM) for 48–72 h (necrosis) and 24–48 h (apoptosis). Necrotic changes were estimated by propidium iodide (PI) staining (**A**) and apoptotic ones by caspase-3 activity measurement (**B**). The data are presented as mean ± SEM of % of PI-positive cells (**A**) or % of vehicle-treated cells for caspase-3 activity (**B**) from 3–4 independent experiments. ^*^P < 0.05 and ^***^P < 0.001 vs. vehicle-treated cells; ^###^*P* < 0.001 vs. 48 h; ^&&&^*P* < 0.001 vs. 24 h. (**C**) Representative DIC (*differential interference contrast*) images of U87MG cells treated for 72 h with TMZ (800 µM), **6** (10 and 30 µM), **4** (30 µM) and **7** (30 µM). Apoptotic cells are indicated by arrows (TMZ) and necrotic ones by asterisks (**6**, **4**, and **7**); for **6**, at a concentration of 30 µM, almost total cell damage is observed.

To identify the most cytotoxic compounds (**6**, **7**, and **4**), we performed mechanistic studies in U87MG cells. First, we measured parameters of necrotic and apoptotic cell death using PI staining and caspase-3 activity assay, respectively. Two-way ANOVA analysis showed that TMZ at concentration 800 µM and after 72 h modestly (7%) but significantly increased the number of PI-positive nuclei (Fig. 5A). The compound **6** in concentration- and time-dependent manner elevated the number of necrotic nuclei. The highest effect was observed for concentration 30 µM after 48 h (25%) and 72 h (55%) (Fig. 5A). We observed a relatively moderate increase in the number of PI-positive cells after incubation with **7** at concentration 10 µM after 48 h (11%) and at 10 and 30 µM after 72 h (5 and 12%, respectively) (Fig. 5A). For **4** we found a relatively small increase in the number of necrotic cells (5%) only after 72 h (Fig. 5A). This effect was very similar to TMZ (800 µM) effect. Two-way ANOVA analysis of caspase-3 activity data demonstrated a concentration- and time-dependent increase in this enzyme activity only by TMZ (400 and 800 µM after 48 h) (Fig. 5B). We did not find any significant increase in caspase-3 activity after 24–48 h of treatment with **6** (3–30 µM), **4** (30 µM) and **7** (3–30 µM) (Fig. 5B). There could be also observed morphological differences between cytotoxic action of TMZ or as shown by light microscopy imaging (Fig. 5C). After 72 h incubation of U87MG cells with TMZ (800 µM) were observed apoptotic bodies (small rounded cells with not damaged cell membranes) being a result of apoptosis activation, whereas after **6** (10 µM), **7** (30 µM) and **4** (30 µM) exposure rather necrotic changes were observed visualized as cells with blurry cell boundaries and with tendency for forming syncytia (Fig. 5C). The 6 at concentration of 30 µM evoked almost total cell damage (Fig. 5C).

Next, using various pharmacological inhibitors, we tested several intracellular mechanisms that could be responsible for cell damage induced by **6**, **7**, and **4**. First we tested the effect of antioxidant - N-acetyl-cysteine (NAC, 1 mM), caspase-3 inhibitor - Ac-DEVD-CHO (Ac, 20 µM), necroptosis inhibitor – necrostatin-1 (Nec-1, 20 µM) and ferroptosis inhibitor – ferrostatin-1 (Fer-1, 20 µM) and none of the tested mechanistic compounds changed the extent of cell damage induced by **6**, **7** and **4** after 24–48 h of treatment (Table S1).

In the second part we tested the effect of ATM kinase inhibitor - KU55933 (KU, 1 µM), mTOR inhibitor - rapamycin (Rap, 0.1 µM), JNK inhibitor - SP600125 (JNKi, 10 µM), ERK1/2 inhibitor - PD98052 (PD, 20 µM) or PI3-K/Akt inhibitor LY294002 (LY, 10 µM) on the spirohydantoin derivatives-mediated cytotoxic action. None of the tested inhibitors attenuated the cell damage induced by **6**, **7** and **4** (Table 3). Interestingly, we found that mTOR inhibitor Rap alone decreased cell viability after 24 and 48 h and increased the cell damage induced by **6** after 24 h, **7** after 24 and 48 h and **4** after 48 h (Table 3). Moreover, ERK 1/2 inhibitor PD98052 alone decreased cell viability in U87MG cells after 24 h and increased the cell damage induced by **7** and **4** after 24 and 48 h (Table 3). The PI3-K/Akt inhibitor LY294002 alone decreased cell viability in U87MG cells after 48 h and increased the cell damage induced by **7** after 24 and 48 h and **4** after 48 h (Table 3). It should be noted that KU55933, after 24 h, and JNKi, after 24 and 48 h, alone significantly increased cell viability, pointing to rather pro-survival effects of these inhibitors (Table 3).

**Table 3:** The effect of ATM kinase inhibitor, mTOR inhibitor, JNK inhibitor, ERK1/2 inhibitor or PI3-K/Akt inhibitor on cell damage induced by compounds **6**, **7** and **4** in U87MG cells.

Group	24 h	48 h
control+veh	100.00 ± 0.00	100.00 ± 0.00
**6**	41.43 ± 2.54 ^***^	22.94 ± 2.11 ^***^
**6** + KU	37.28 ± 1.69 ^***^	26.39 ± 1.65 ^***^
**6** + Rap	27.21 ± 2.06 ^***, #^	12.79 ± 4.76 ^***^
**6** + JNKi	39.29 ± 2.87 ^***^	24.60 ± 1.64 ^***^
**6** + PD	32.37 ± 2.29 ^***^	17.39 ± 1.12 ^***^
**6** + LY	32.82 ± 2.84 ^***^	16.92 ± 1.54 ^***^
**7**	69.74 ± 3.91 ^***^	64.45 ± 4.14 ^***^
**7** + KU	66.78 ± 6.29 ^***^	57.00 ± 6.94 ^***^
**7** + Rap	52.18 ± 5.93 ^***, #^	44.29 ± 4.76 ^***, #^
**7** + JNKi	68.19 ± 4.85 ^***^	57.51 ± 4.86 ^***^
**7** + PD	43.37 ± 1.43 ^***, ###^	38.35 ± 4.04 ^***, ###^
**7** + LY	52.61 ± 2.47 ^***, #^	44.83 ± 3.27 ^***, #^
**4**	60.25 ± 2.56 ^***^	57.55 ± 4.81 ^***^
**4** + KU	57.37 ± 4.08 ^***^	48.66 ± 2.28 ^***^
**4** + Rap	50.05 ± 5.34 ^***^	40.48 ± 1.32 ^***, #^
**4** + JNKi	60.33 ± 6.26 ^***^	51.03 ± 3.78 ^***^
**4** + PD	41.93 ± 1.96 ^***, ##^	36.53 ± 0.64 ^***, ##^
**4** + LY	51.11 ± 4.16 ^***^	38.41 ± 2.13 ^***, #^
KU	113.99 ± 9.09 ^*^	109.56 ± 8.97
Rap + veh	70.92 ± 8.44 ^***^	59.29 ± 6.51 ^***^
JNKi	121.95 ± 2.90 ^***^	115.48 ± 11.85 ^*^
PD	82.69 ± 3.01 ^**^	94.04 ± 7.15
LY + veh	90.00 ± 6.59	81.63 ± 7.81 ^*^

The U87MG cells were pre-treated for 30 min with ATM kinase inhibitor - KU55933 (KU, 1 µM), mTOR inhibitor - rapamycin (Rap, 0.1 µM), JNK inhibitor SP600125 (JNKi, 10 µM), ERK1/2 inhibitor PD98052 (PD, 20 µM) or PI3-K/Akt inhibitor LY294002 (LY, 10 µM) followed by 24–48 h exposure to 30 µM of compounds **6**, **7** and **4**. Cell viability was estimated by the MTT reduction test. The data were normalized to vehicle-treated cells (H_2_O/DMSO) and are presented as mean ± SEM from 3 to 7 independent experiments. ^*^P < 0.05, ^**^P < 0.01 and ^***^P < 0.001 vs. vehicle-treated cells; ^#^*P* < 0.05, ^##^*P* < 0.01 and ^###^*P* < 0.001 vs. **6**, **7** or **4**-treated cells.

In the third part, we tested the effect of calpain inhibitors – MDL28170 (MDL, 20 µM) or calpeptin (Calp, 20 µM), and Cathepsin D inhibitor – pepstatin A (PsA, 0.3 µM) on the **6-**, **7-** or **4-**mediated cytotoxic action. We found that these inhibitors, when given alone to U87MG cells for 24 and 48 h, significantly decreased cell viability. Moreover, Calp increased **6**-, **7**- and **4**-evoked cell damage after 24 and 48 h whereas MDL28170 increased **7**-evoked cell damage after 24 and 48 h and **4**-evoked cell damage after 24 h (Table 4). PsA did not change the extent of cell damage induced by **6**, **7**, and **4** after 24 or 48 h of treatment (Table 4).

**Table 4:** The effect of calpain and cathepsin D inhibitors on cell damage induced by compounds **6**, **7** and **4** in U87MG cells.

Group	24 h	48 h
control+veh	100.00 ± 0.00	100.00 ± 0.00
**6**	36.24 ± 3.47 ^***^	20.73 ± 2.35 ^***^
**6** + MDL	28.55 ± 1.55 ^***^	10.54 ± 2.87 ^***^
**6** + Calp	24.38 ± 1.68 ^***, #^	5.2 ± 2.88 ^***, #^
**6** + PsA	38.72 ± 2.97 ^***^	25.24 ± 2.08 ^***^
**7**	68.08 ± 3.69 ^***^	66.20 ± 3.99 ^***^
**7** + MDL	51.85 ± 2.77 ^***, ##^	47.84 ± 3.06 ^***, #^
**7** + Calp	46.12 ± 5.37 ^***, ###^	44.57 ± 2.51 ^***, ##^
**7** + PsA	65.45 ± 4.92 ^***^	58.49 ± 4.30 ^***^
**4**	60.25 ± 2.56 ^***^	59.48 ± 4.50 ^***^
**4** + MDL	47.73 ± 3.39 ^***, #^	45.89 ± 3.08 ^***^
**4** + Calp	41.42 ± 4.13 ^***, ##^	37.79 ± 2.43 ^***, ##^
**4** + PsA	54.47 ± 1.91 ^***^	51.42 ± 2.56 ^***^
MDL + veh	86.32 ± 2.40 ^**^	77.69 ± 3.62 ^**^
Calp	81.38 ± 4.15 ^***^	69.15 ± 20.29 ^***^
PsA + veh	80.49 ± 3.58 ^***^	83.00 ± 6.07 ^*^

The U87MG cells were pre-treated for 30 min with calpain inhibitors – MDL28170 (MDL, 20 µM) or calpeptin (Calp, 20 µM), and Cathepsin D inhibitor – pepstatin A (PsA, 0.3 µM) followed by 24 and 48 h exposure to 30 µM of **6**, **7** and **4**. Cell viability was estimated by the MTT reduction test. The data were normalized to vehicle-treated cells (H_2_O/DMSO) and are presented as mean ± SEM from 3 to 8 independent experiments. ^*^P < 0.05, ^**^P < 0.01 and ^***^P < 0.001 vs. vehicle-treated cells; ^#^*P* < 0.05, ^##^*P* < 0.01 and ^###^*P* < 0.001 vs. **6**, **7** or **4**-treated cells.

### Effects of combined treatment of compounds **4**, **6** or **7** with TMZ in U87MG cells

**Fig. 6:**
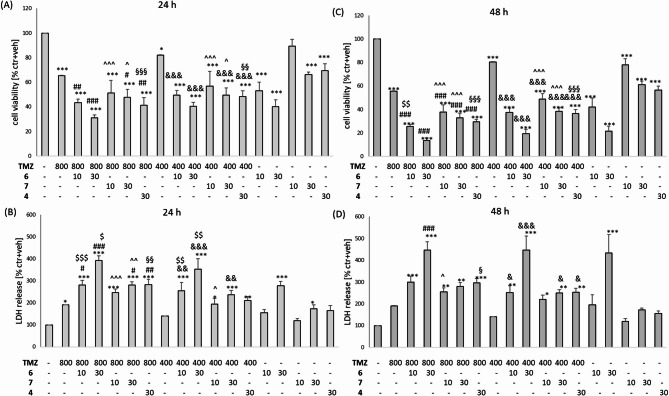
The cytotoxic effect of combined treatment with temozolomide (TMZ) and compounds **6**, **7** and **4** in U87MG. The cells were treated with TMZ (800 and 400 µM) or compounds **6**, **7** and **4** for 24 h (A, B) and 48 h (C, D). Cell viability was estimated by the MTT reduction test (A, C) and cytotoxicity by the LDH release assay (B, D). The data were normalized to vehicle-treated cells and are presented as mean ± SEM from 3 independent experiments. ^*^P < 0.05, ^**^P < 0.01 and ^***^P < 0.001 vs. vehicle-treated cells; ^#^*P* < 0.05, ^##^*P* < 0.01and ^###^*P* < 0.001 vs. TMZ 800s; ^&^*P* < 0.05, ^&&^*P* < 0.01 and ^&&&^*P* < 0.001 vs. TMZ 400; ^$^*P* < 0.05, ^$$^*P* < 0.01 and ^$$$^*P* < 0.001vs. **6** at particular concentration; ^^^*P* < 0.05, ^^^^*P* < 0.01 and ^^^^^*P* < 0.001 vs. 7 at particular concentration; ^§^*P* < 0.05, ^§§^*P* < 0.01 and ^§§§^*P* < 0.001 vs. **4**.

Compound **6** at concentrations 10 and 30 µM significantly exaggerated the TMZ 800 or 400 µM cell-damaging effects as confirmed at both studied time points (24 and 48 h) with the MTT reduction assay (Figs. 6A, C) and after 24 h in the LDH release assay (Fig. 6B) when compared to relevant TMZ groups. In the LDH assay after 48 h we found a significant increase in the LDH level when 30 µM of **6** was combined with TMZ 800 or 400 µM and for 10 µM of **6** with TMZ 400 µM (Fig. 6D) when compared to the relevant group treated only with TMZ. However, the effect of combined treatment of this compound with TMZ versus the cytotoxic effects mediated by **6** alone was significantly higher for 10 µM concentration of this compound combined with TMZ 800 µM after 48 h in the MTT reduction assay (Fig. 6C) and for both concentrations of **6** (10 and 30 µM) combined with TMZ 800 or 400 µM after 24 h in the LDH release assay (Fig. 6B).

Compound **7** at concentration 30 µM significantly exaggerated the TMZ 800 or 400 µM cell-damaging effects after 24 h in the MTT assay (Fig. 6A) and both concentrations (10 and 30 µM) increased the TMZ (800 and 400 µM)-induced decrease in cell viability after 48 h (Fig. 6C) when compared to TMZ effects alone. In the LDH assay we found the increase in cytotoxicity after 24 h of treatment of 30 µM of compound **7** with TMZ 800 and 400 µM (Fig. 6B) and after 48 h for 30 µM of compound **7** in combination with TMZ 400 µM when compared to the effect of TMZ alone at particular concentrations (Fig. 6D). When compared the effects of combined treatment of compound **7** with TMZ in comparison to the effect of **7** alone in the MTT assay we detected a significant exaggeration of cell damage for: 10 and 30 µM of compound **7** plus TMZ 800 µM or TMZ 400 µM after 24 (Fig. 6A) and 48 h (Fig. 6C). The same comparison made for the LDH assay data demonstrated a significant increase in cytotoxicity for: 10 and 30 µM of **7** and TMZ 800 µM and 10 µM of **7** and TMZ 400 µM after 24 h (Fig. 6B); 10 µM of **7** and TMZ 800 µM after 48 h (Fig. 6D).

**Fig. 7:**
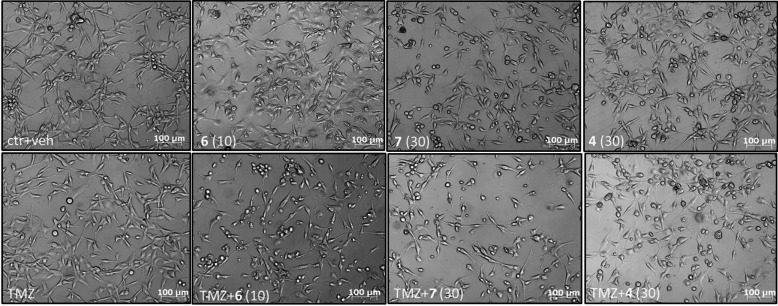
Representative DIC (differential interference contrast) images of U87MG cells treated for 48 h with **6** (10 µM), **7** (30 µM) and **4** (30 µM) alone and in combination with TMZ (800 µM).

Compound **4** (30 µM) significantly exaggerated the TMZ 800 or 400 µM cell-damaging effects after 24–48 h in the MTT assay when compared to the effects of TMZ alone (Fig. 6A, B). In the LDH assay, the same comparison demonstrated a significant increase in cytotoxicity for **4** plus TMZ 800 µM after 24 h (Fig. 6B) and plus TMZ 400 µM after 48 h (Fig. 6D). The comparison of the effects of combined treatment of **4** with TMZ versus the effects of **4** alone showed in the MTT assay a significantly higher cell-damaging effect of combined treatment of **4** with TMZ 800 and 400 µM at both studied time points (Fig. 6A, C). However, in the LDH assay, a significant increase in cytotoxicity was detected only for the combination of **4** with TMZ 800 µM after 24 h (Fig. 6B) and 48 h (Fig. 6D). The synergistic cytotoxic effects of TMZ (800 µM) and **6** (10 µM), **7** (30 µM) and **4** (30 µM) were also confirmed by light microscopy imaging (Fig. 7).

Moreover, by using the PI staining method, we demonstrated at the level of the number of necrotic cells a synergistic effect of compound **6** at concentrations of 10 and 30 µM with TMZ 800 µM in comparison to the effects of TMZ alone after 48 h (Fig. S5A) and 72 h (Fig. S5B). Moreover, these combinations were also significant at both time points when compared to the effects of compound **6** alone at particular concentrations (Fig. S5A, B). For compound **7** and compound **4** we observed that their combination with TMZ 800 µM significantly increased the number of necrotic cells after 48 and 72 h when compared to the effects of TMZ alone, but these combinatory treatments were significant only after 72 h when compared to the effects of **7** or **4** alone (Fig. S5A, B).

At the level of caspase-3 activity, we found a significant increase in this enzyme activity for U87MG cells treated for 48 h with TMZ 400 µM plus **7** (30 µM), TMZ 800 µM, TMZ 800 µM plus **7** (30 µM) and TMZ 800 µM plus **4** (30 µM) when compared to vehicle-treated cells (Fig. S5C). Moreover, we showed a significantly higher activation of caspase-3 in cells treated with **7** ( 30 µM) with TMZ 800 µM or TMZ 400 µM when compared to the effects of **7** alone (Fig. S5C).

Using a BrdU staining method and flow cytometry after 48 h of U87MG cell treatment with **6** (3 and 10 µM), **7** ( 10 and 30 µM) and **4** ( 10 and 30 µM) alone and in combination with TMZ 400 µM we found that none of the tested compounds affected the proliferation rate compared to vehicle-treated cells (Fig. S5D). We found a significant reduction in cell proliferation for experimental groups: TMZ 400 µM and for its combinations with 3 µM of **6** and 30 µM of **4**, however, they were statistically different only to vehicle-treated cells but not when compared to effects of TMZ or particular tested compounds alone (Fig. S5D).

### Effects of combined treatment of compounds **4**, **6** and **7** with TMZ in A172 and U138MG cells

In A172 cells, we found in the MTT reduction assay a significant exaggeration of cell damage induced by TMZ (400 µM) by compound **6** (10 µM) and **7** (30 µM) after 24 h and for all tested compounds after 48 h, when compared to the effects of TMZ alone (Table S3). When comparing the effects of combined treatment of **6**, **7** or **4** with TMZ in comparison to the effect of a particular tested compound alone in the MTT assay, we detected a significant exaggeration of cell damage for 30 µM of compound **7** and compound **4** plus TMZ after 24 h and for all tested compounds after 48 h (Table S3). In the LDH release assay, we observed a significantly increased level of cytotoxicity by **7** after 24 h and by **6**, **7**, and **4** after 48 h when combined with TMZ in comparison to TMZ alone (Table S3). In comparison to the effects of particular tested compounds alone in the LDH test, the combinations of **7** or **4** with TMZ after 24 h and **4** with TMZ after 48 h were significantly more cytotoxic (Table S3).

In U138MG cells, we found in the MTT reduction assay a significant exaggeration of cell damage induced by TMZ (400 µM) by compound **6** at two tested concentrations (10 and 30 µM), **7** ( 30 µM) and **4** ( 30 µM) after 48 h when compared to the effects of TMZ alone (Table S4). When comparing the effects of combined treatment of **6**, **7**, and **4** compounds with TMZ in comparison to the effect of a particular tested compound alone in the MTT assay, we detected a significant exaggeration of cell damage for compound **7** plus TMZ after 48 h (Table S4). In the LDH release assay, we observed a significantly increased level of cytotoxicity by 30 µM of **6** after 24 and 48 h, and by **7** after 48 h when combined with TMZ in comparison to TMZ alone (Table S3). In comparison to the effects of a particular tested compound alone in the LDH test, only the combination of **7** with TMZ after 48 h was significantly more cytotoxic (Table S4).

### Radioligand binding assays

For all the investigated compounds (**1–8)**, radioligand binding assays were performed in order to estimate their affinity for dopaminergic D_2_R and homologous serotoninergic receptors 5-HT_1A_R, 5-HT_2A_R and 5-HT_7_R. Results are presented in Table 5. The highest (*K*_*i*_ < 100 nM) affinity to D_2_R **5**. It should be noted that the compounds with the highest affinity to D_2_R, **3**, and **7** also had the highest affinity to 5-HT_1A_R.

For the compounds with the most promising anticancer profile **4**, **6**, and **7**, the ability to inhibit also D_4_R was tested in the presence of a D_4_ antagonist (Table 6). The highest inhibitory effect on D_4_R was demonstrated by **7**, medium by **4**, and the lowest by **6**.

**Table 5:** Radioligand binding assays results for the investigated hydantoin-phenylpiperazines **1–8.**

Compound	K_i_ ± SD [nM]^a^			
	D_2_*R*	5-HT_1A_*R*	5-HT_2A_*R*	5-HT_7_*R*
**1**	441 ± 51	378 ± 63	2994 ± 682	1870 ± 352
**2**	383 ± 44	69 ± 8	2719 ± 371	686 ± 51
**3**	**56** ± 9	6 ± 2	5906 ± 1372	633 ± 109
**4**	300 ± 74	38 ± 5	847 ± 104	190 ± 28
**5**	973 ± 193	442 ± 71	9256 ± 1987	3898 ± 3898
**6**	133 ± 17	198 ± 34	1266 ± 265	1414 ± 173
**7**	**13** ± 4	32 ± 4	716 ± 85	225 ± 19
**8**	119 ± 14	347 ± 39	11,360 ± 2654	3798 ± 527

^a^The inhibition constants (*K*_i_) were calculated using the Cheng–Prusoff equation. Data expressed as the mean ± SD of three independent experiments carried out in duplicate. Values of K_i_ below 100 nM are bolded.

**Table 6:** The percentage of inhibition of D_4_R at 1 µM for compounds **4**, **6** and **7**.

Compound	4	6	7
D_4_R*	73	30	95

* % inhibition of control specific binding at 1.0E-06 M.

### Expression of dopamine receptors from D_2_-like family in U87MG, A172 and U138MG cell lines

Some previous data reported the presence of D_2_R receptors in the cell lines chosen by us for the anti-GBM drug screening^44,45^. In order to relatively assess the expression level of these receptors, as well as other members of the D_2_-like family (D_3_R, D_4_R) in various GBM cell types, we used a quantitative JESS capillary-based western blotting system (Protein Simple Jess, Biotechne). We compared the expression level of studied receptors in U87MG, A172 and U138MG cells with their expression in human neuroblastoma SH-SY5Y cells, a widely used and accepted model of dopaminergic cells^46^, which served for us as a positive control. Although the studied GBM cell lines differed between

**Fig. 8:**
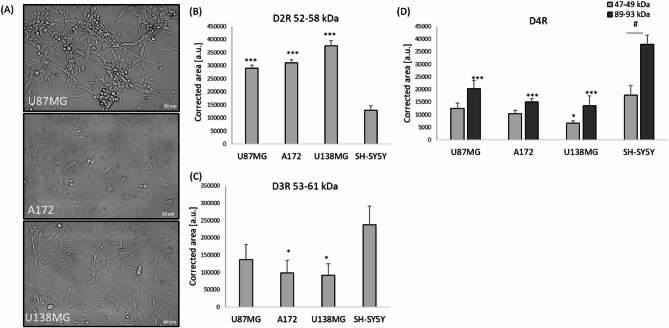
**A** Representative DIC images of U87MG, A172 and U138MG cells, **B–D** expression of D_2_R (B), D_3_R (**C**) and D_4_R (**D**) in U87MG, A172 and U138MG GBM cells. Neuroblastoma SH-SY5Y cells were used as a reference cell line with a dopaminergic phenotype. Protein level was measured by quantitative JESS capillary-based western blotting system (Protein Simple Jess, Biotechne). Data are expressed in arbitrary units as mean ± SEM of corrected area from two independent experiments, each with triplicates. ^*^P < 0.05 and ^***^P < 0.001 vs. SH-SY5Y cells; ^#^*P* < 0.05 monomer vs. dimer of D_4_R.

themselves at morphological level (Fig. 8A), proliferation rate (the highest in A172 cells, medium in U87MG, and the lowest in U138MG, as deduced by microscopic observations during cell passages) and metabolic activity (the highest in U87MG, medium in U138MG and the lowest in A172, as could be concluded from the MTT assay), at the level of D_2_-like receptors we did not reveal any significant changes between particular GBM cell lines (Fig. 8). However, we observed a significantly higher expression of D_2_R in all studied GBM cell lines when compared to their expression in SH-SY5Y cells (Fig. 8B). In case of D_3_R and D_4_R expression, their level was relatively lower in GBM cell lines when compared to SH-SY5Y cells (Fig. 8C, D). It should be noted that in case of D_4_R, we observed a monomeric and dimeric forms, where the latter one was predominating which was statistically evidenced by two-way ANOVA analysis for SH-SY5Y cells (a significantly higher level of dimer vs. from monomer) (Fig. 8D). The original data from expression of all D_2_-like receptors as well total protein measurements are available at Supplementary materials_Part 2 file.

### Molecular modelling

**Fig. 9:**
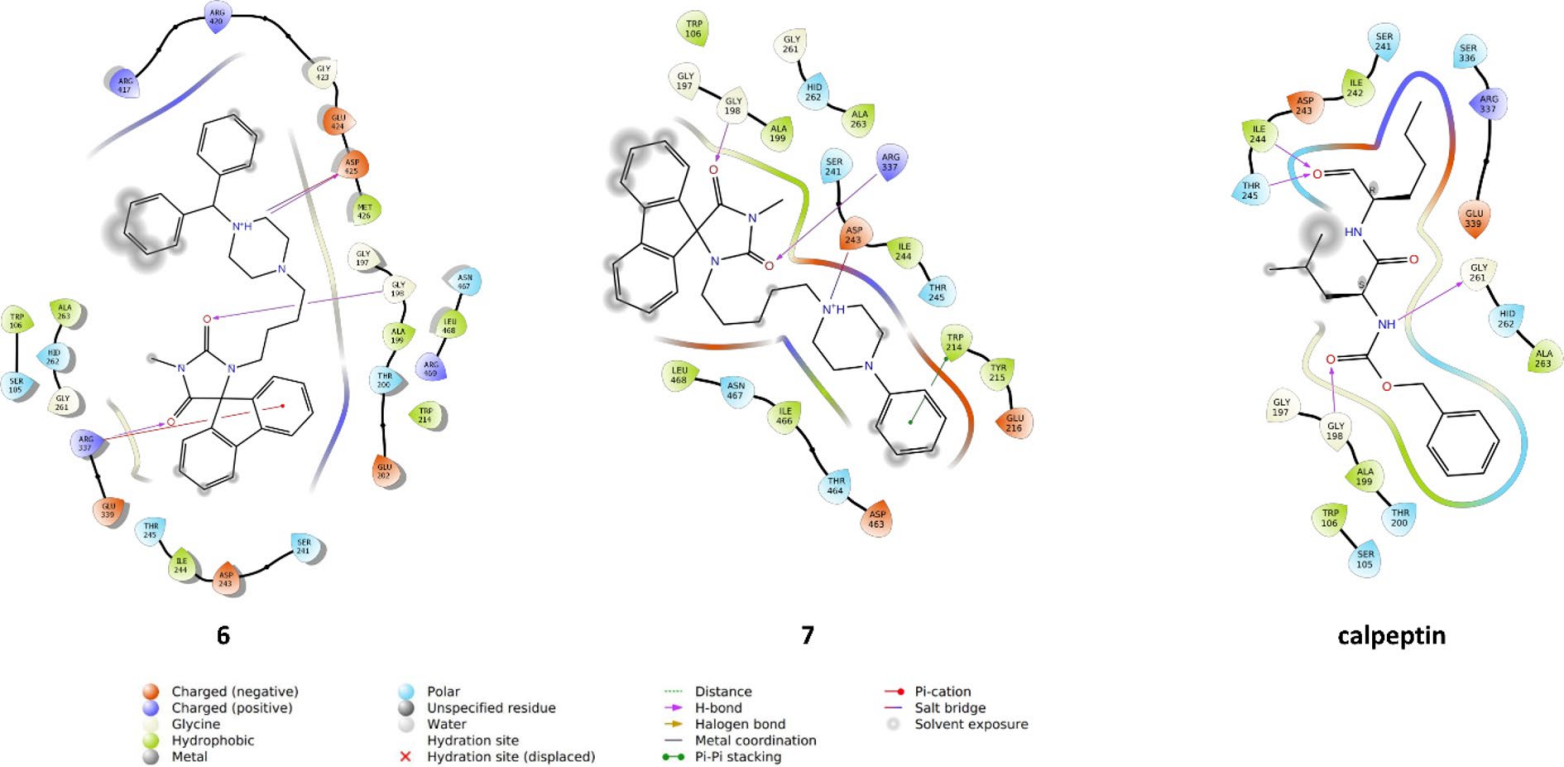
Ligand-protein interaction diagrams obtained for ligand µ-calpain complexes for calpeptin and compound **7**.

**Fig. 10:**
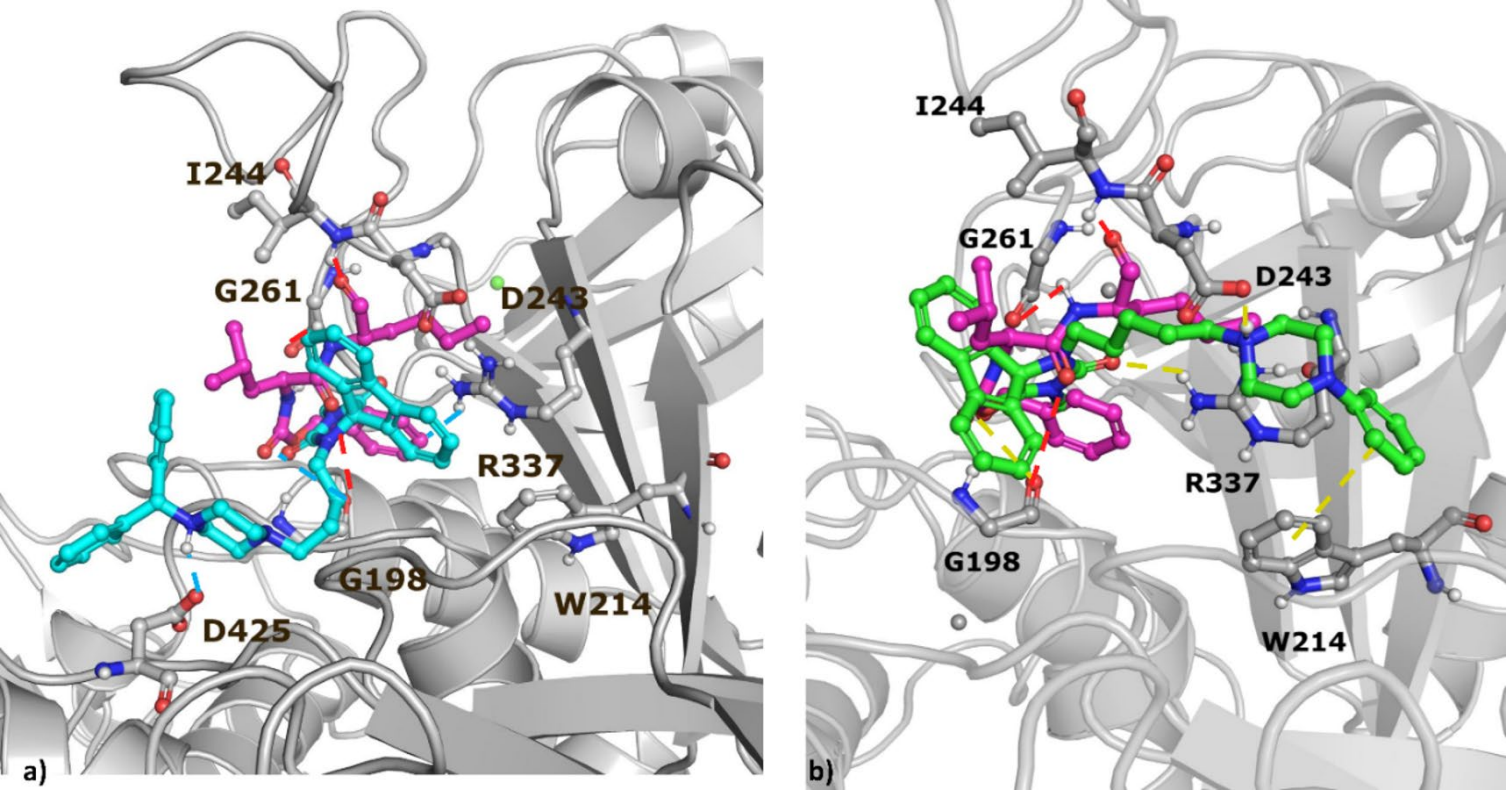
Docking poses obtained for the examined compounds: **6** (cyan), **7** (green), calpeptin (magenta).

As the most promising compounds with anticancer activity **6**, **7** and **4** showed synergism with calpain inhibitors, and moreover, we noticed that the hydantoin moiety might be a bioisoster of the peptide bond present in reported calpain inhibitors^47,48^. We evaluated the potential of selected compounds to inhibit µ-calpain in silico via docking with the use of the crystal structure 3BOW. Despite the examined compounds, calpeptin, the potent calpain inhibitor was docked as a reference compound. The docking results indicated potential ligand-protein interactions for all compounds that displayed activity with calpeptin (compounds **4**, **6**, and **7**); however, in our analysis, we focus on the most promising compounds **6** and **7** (as the second most promising compound and representative of a linear chemotype in contrast to the branched structure of **6**). The results are presented in the form of the ligand-protein interaction diagrams (Fig. 9) and the docking poses visualizations (Fig. 10).

Examination of the docking poses of **6**, **7** and calpeptin indicated a lot of similarities in their conformations in the binding site. The fluorene and hydantoin moieties of **6** and **7** occupy similar region of the µ-calpain binding site, as calpeptin. It is also reflected by the similar set of interactions formed by these compounds, in particular, the hydrogen bond between the carbonyl group of hydantoin and GLY198 (both in the case of **6** and **7**), which is also observed in the calpeptin-µ-calpain complex between the carbonyl from the peptide bond and GLY198. This observation can be an indication of the equivalent role of the hydantoin in the examined compounds and the peptide bond of calpeptin. In addition, the second carbonyl from the hydantoin of **6** and **7** forms a second hydrogen bond, this time with ARG337. In the case of **6**, the interaction with ARG337 is further enhanced by the pi-cation contact with one of the benzene rings of the fluorene, which might be an important contribution to the enhanced biological activity of this compound. Moreover, thanks to the presence of the piperazine moiety in **6** and **7**, its protonated nitrogen forms a charge-assisted hydrogen bond with the aspartic acid residues (ASP425 and ASP243, respectively), and the attached phenyl in compound **7** is part of the pi-pi interaction with TRP214. All these contacts provide a strong and stable fitting of **6** and **7** in the µ-calpain binding pocket. On the other hand, the peptide bond, which makes contact with GLY198 in calpeptin, also forms a hydrogen bond with GLY261 via the NH group. Calpeptin is fixed in the µ-calpain binding pocket via two additional hydrogen bonds with ILE244 and THR245.

The energy of docking poses was assessed via the analysis of the Glide emodel parameter. It combines the main energetic components of the protein-ligand complex, including van der Waals and Coulombic interaction energies as well as the internal strain energy of the ligand, together with the GlideScore contribution. As a result, Emodel provides a more physically meaningful estimate of the relative stability of docking poses compared to scoring-only metrics. Compound **6** exhibited the most favorable energy (–81.70 kcal/mol), indicating the formation of the most stable docking pose among the analyzed molecules. In contrast, compound **7** and calpeptin showed substantially higher (less negative) Emodel values, − 59.541 kcal/mol and − 59.203 kcal/mol, respectively. The analysis of these docking data suggest that the interactions formed by **6 **within the binding site are energetically more optimal, leading to a more stable complex compared to both the reference inhibitor and **7**. The similar Emodel values of **7** and calpeptin indicate comparable interaction strengths and pose stabilities for these two molecules.

**Fig. 11:**
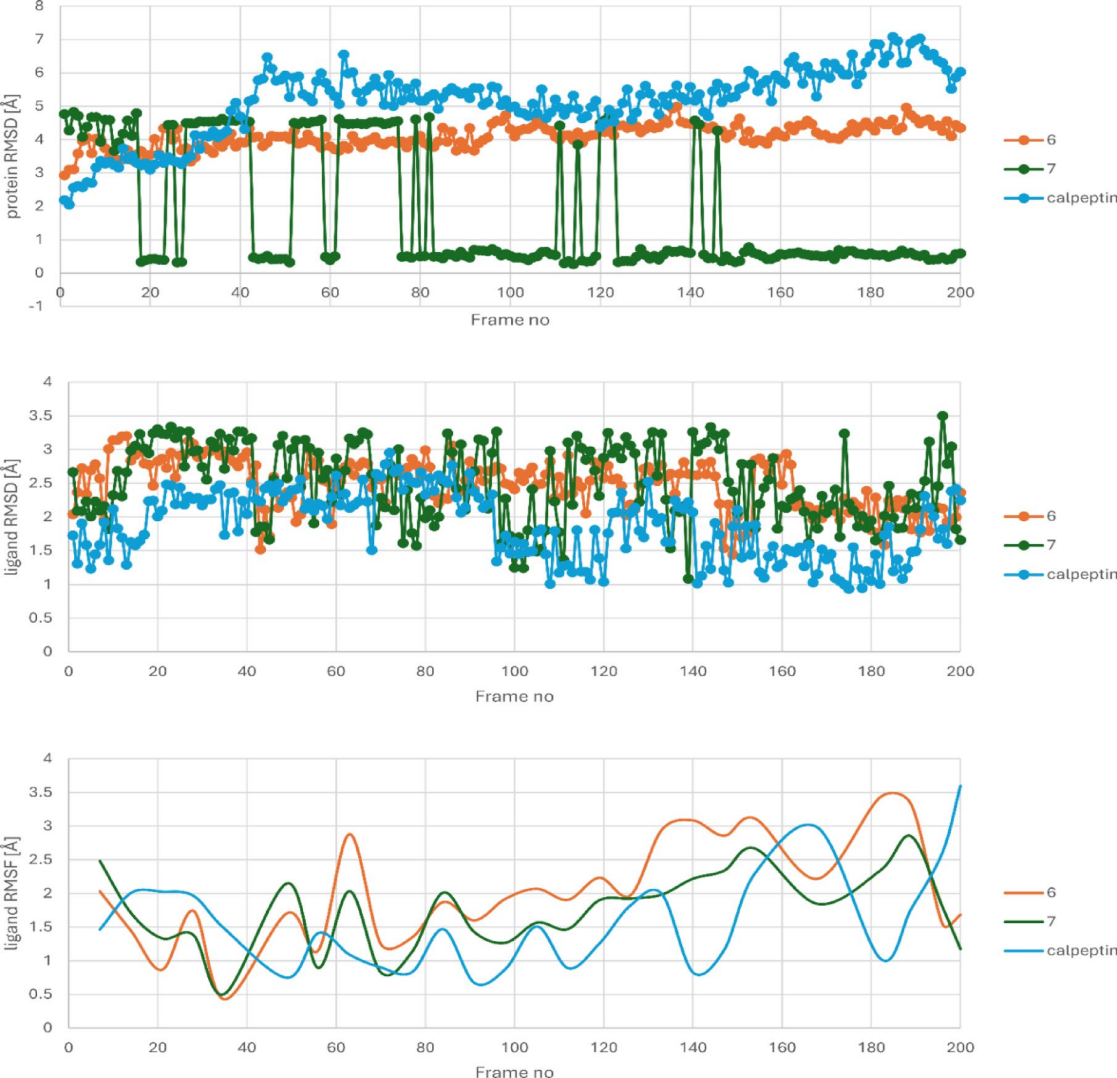
Analysis of ligand and protein RMSD values and ligand RMSF values obtained during MD simulations studies.

In order to validate the obtained docking poses, 200-ns molecular dynamics (MD) simulations were performed. The docking poses of compound **7** remained consistent and stable throughout the entire simulation. In contrast, the orientation of compound **6** and calpeptin within the binding pocket was only periodically stable - after the initial several 60 ns of MD, calpeptin slightly changed its orientation during the following 100 ns, before undergoing another positional rearrangement toward the end of the simulation; whereas **6** slightly changed its orientation after initial 20 ns of simulations and remained stable in this position (Fig. S6). Overall, compounds **6** and **7** were more stably anchored within the protein binding site, which further supports their intended biological activity; analysis of ligand and protein RMSD, as well as ligand RMSF is provided on Fig. 11. It is important to note that the relatively high protein RMSD values observed in these simulations stem from the intrinsic structural properties of µ-calpain, which possesses an open and solvent-accessible architecture adapted to accommodate large peptide substrates, in contrast to the compact and rigid binding pockets typical of many classical enzymes. In addition, it contains numerous partially ordered, acidic residue-rich segments which undergo pronounced conformational rearrangements in MD simulations and therefore substantially contribute to global RMSD values without indicating instability of the protein-ligand complexes. Additional analysis of ligand–protein complex stability was performed by monitoring the radius of gyration and solvent-accessible surface area (SASA). Both parameters remained stable throughout the simulation and did not vary by more than several percentage points over the entire simulation time (Figs. S8, S9). In addition, hydrogen bond interaction patterns were monitored throughout the simulations. For compound **6**, a hydrogen bond with ALA101 was present for 74% of the simulation time, while a hydrogen bond with HIS262 occurred for 61% of the simulation time. For compound **7**, hydrogen bonds with ARG337 and ASN467 were maintained for 75% and 52% of the simulation time, respectively. In the case of calpeptin, hydrogen bonds with ALA101, GLY198, ALA252, and GLY261 were observed for 48%, 44%, 53%, and 39% of the simulation time, respectively, which supports the qualitative analysis of the MD data carried out previously.

Therefore, given the open and conformationally flexible architecture of µ-calpain and the presence of intrinsically flexible regions, for µ-calpain, as well as other similar proteins, RMSD should not be treated as a standalone metric of ligand-protein complex stability. Instead, RMSF, radius of gyration, SASA, hydrogen-bond analysis, and ligand-protein contact profiles should also be taken into account to provide a more comprehensive evaluation of complex stability.

For the ligand–protein complexes minimized using the MD-based protocol, the binding energies were estimated by combining molecular mechanics energies with implicit solvation models, applying the Molecular Mechanics – Generalized Born Surface Area (MM-GBSA) approach. The results were analyzed not only through direct comparison of the predicted ΔG_bind values, but also by examining the individual energetic components contributing to the overall binding energy (Fig. S7). The obtained MM-GBSA results revealed comparable binding affinities among the examined compounds. The most favorable predicted binding free energy (ΔG_bind = − 52.7 kcal/mol) was observed for calpeptin, closely followed by **7** (− 51.2 kcal/mol) and **6** (− 48.9 kcal/mol). Although the overall binding energies are similar, the energetic decomposition indicates distinct interaction profiles. Compounds **6** and **7** exhibit strong electrostatic (Coulombic) stabilization, considerably higher than that observed for calpeptin. In contrast, calpeptin benefits from more favorable van der Waals and lipophilic interactions. Collectively, these results suggest that all three ligands are capable of binding effectively to µ-calpain.

### Prediction of ability to cross BBB

Taking into account the intended pharmacological application of the investigated compounds, their potential to cross the BBB was evaluated. To this end, QikProp predictions from the Schrödinger Suite were performed, focusing on the QPlogBB parameter, which estimates the brain–blood partition coefficient for orally administered drugs and reflects their ability to penetrate the BBB. The recommended QPlogBB range for compounds capable of effective BBB permeation spans from − 3.0 to + 1.2. The tested compounds — calpeptin, **4**, **6**, and **7** — were all predicted to possess BBB permeability within this range, with calculated QPlogBB values of − 1.511, 0.029, 0.012, and − 0.161, respectively.

## Discussion

The present study demonstrated that among the eight tested 5-spirofluorenohydantoin derivatives, **6**, **7** and **4** could be pointed as the compounds with the highest cell-damaging activity towards GBM cells based on: (a) investigation in U87MG cells under cell proliferation favourable and inhibitory conditions); (b) relatively low (**6** and **7**) or absent (**4** ) cytotoxicity towards human skin fibroblasts (CI > 7); (c) relatively good solubility (dissolved in H_2_O/DMSO mixture) (Table 1). These three agents were also cytotoxic to A172 cells (Table 2), with a similar order of effectiveness to U87MG cells, **6** > **7**>**4** (Table 1), and A172 cells appeared to be more sensitive to **6**,** 7**, and TMZ toxicity when compared to U87MG cells (lower IC_50_). In the U138MG cell, we did not observe any cell-damaging effect of **4**, and observed cytotoxic effect of **6** and **7** was relatively weaker (higher IC_50_) than found in A172 (Table 4) or U87MG cells (Table 2). Heterogeneity in treatment conditions, compound concentrations, and cell lines across experiments were introduced intentionally to address distinct experimental aims within each model system. Serum starvation was applied in selected assays to enable discrimination between cytostatic and cytotoxic effects. Solvents were adjusted according to solubility constraints and supported by preliminary viability assessments to ensure reliable and interpretable measurements. The time checkpoint differed regards on particular assays as when investigating cell death mechanisms mediated by various compounds we should take into account it’s their dynamic character, thus, some earlier steps, like caspase-3 activity, should be monitored earlier (in our case after 24 and 48 h), whereases later ones, like necrotic nuclei, should be monitored later on (48 and 72 h). While this experimental heterogeneity limits strict quantitative comparisons between datasets, each experiment was designed to be internally consistent and biologically interpretable within its specific context. This approach is consistent with current perspectives emphasizing that disease-relevant and translationally meaningful preclinical assays often require context-specific model design rather than uniform experimental conditions across systems.

The observed higher sensitivity of A172 cells to cell-damaging effects of compounds **6**, **7**,** 4** and TMZ when compared to U87MG cells or U138 MG could be explained by a higher proliferation rate of A172 cells, observed during cell passaging, when compared to other used GBM cells (U87MG or U138MG), under the same cell culture conditions (DMEM with 10% FBS + 1% S + P). Since the IC_50_ calculation results of the MTT reduction assay do not differentiate between cytotoxic and cytostatic effects of the tested compound, it is highly probable that the higher sensitivity of A172 cells observed in this assay could be a result of higher cytostatic effects of the tested agents in A172 cells. To this end, in the paper by Lee et al.^38^ higher sensitivity of A172 cells to cytotoxic action of curcumin was observed, when compared to U87MG cells, which authors connected with higher autophagy protein expression and higher basal autophagic flux in U87MG cells which made them more resistant to curcumin cytotoxicity when compared to A172 cells. Moreover, the authors observed a lower cytotoxic effect of curcumin when they deprived cells of serum for 6 h. This is in line with our observation, that under serum deprivation conditions IC_50_ of **6**, **7** and **4** increased suggesting lower cytotoxic effect when compared to cells growing and treated in medium with 10% FBS content (Table 2). Another interesting hypothesis explaining the higher vulnerability of A172 to cell-damaging action of the spirohydantoin derivatives is their possible dependence on metabolic status of the GBM cells. It has been reported that U87MG cells are more glycolytic, whereas A172 is based on mitochondrial oxidative phosphorylation^49,50^. Thus in our future work we are planning for the best anti-GBM compounds from the spirohydantoin family to measure metabolic activity (the extracellular flux changes and oxygen consumption rate) with the Seahorse XF96 Analyzer (Agilent). It should be noted that in our study the cytotoxic activity of the most promising agents (**6**, **7** or **4**) was greater than these one observed for clinically used chemotherapeutic TMZ and all of these compounds significantly increased cell damage of GBM cells when combined with TMZ in comparison to the effect of particular compounds given w/o TMZ (Figs. 6 and 7, S5 and Tables S3). Similar findings were from GBM resistant phenotype (U138MG), where TMZ was not effective alone, but when combined with **6**, **7**, or **4**, it significantly reduced cell viability (Table S4). These data point to the potential utility of **6** in monotherapy of GBM, and these three 5-spirofluorenohydantoin derivatives (**6**, **7**, and **4**) as adjuvant therapeutics for GBM when combined with classical chemotherapy (TMZ). It should be noted that cell viability reducing potential of the best acting hydantoin-derived compounds (**6**, **7** and **4**) could be explained as a combination of their cytostatic and cytotoxic effects as evidenced by comparison of their IC_50_ values in U87MG cells growing in cell proliferation favouring (medium with 10% FBS) and inhibitory (medium w/o FBS) conditions (Table 1). It should be noted that almost all the hydantoin-derived compounds tested in this study, with the exception of **2**, demonstrated this mixed cytostatic and cytotoxic activity in U87MG (Table 1). However, when using a specific test for cell proliferation (BrdU labelling), we did not confirm this effect for the best acting compounds (**6**, **7**, and **4**), although TMZ significantly reduced this parameter (Fig. S5D). Thus two scenarios could explain these discrepancies between cell viability and BrdU method: (i) the chosen time for BrdU staining was not sufficient to detect the significant inhibitory effects of investigated hydantoins on cell proliferation or (ii) the results observed in the MTT assay could demonstrate negative impact of tested compounds on cell metabolism which with time will lead to cell death and in this case are not connected with impact on cell proliferation. The calculations of the QPlogBB parameter resulted in satisfactory values for compounds **6**, **7**, and **4**, thus indicating their potential to cross the BBB and be active in vivo.

When considering the cytotoxic mechanisms of compounds **6**, **7** and **4** it could be noticed a significant difference of their action in comparison to TMZ, when the former induced a significant necrotic changes w/o activation of caspase-3, and effector apoptotic protease whereases a reference compound (TMZ) induced significant apoptosis and to extend also lesser necrosis (Fig. 5A, B) which from the time perspective (significant increase after 72 h) could be regarded as a secondary necrosis which under in vitro condition is a results of apoptotic cell death^51,52^. Regarding an involvement of potential mechanisms, which could be responsible for observed cytotoxic activity of **6**, **7** or **4**, we excluded the participation of increased oxidative stress; induction of programmed cell death pathways like apoptosis, necroptosis or ferroptosis; an activation of ATM or JNK pathways since none of the tested inhibitors of these targets inhibited cell damage induced by **6**, **7** or **4** as tested in U87MG cells (Table S2, Table 3). We confirmed an involvement of inhibition of pro-survival pathways MAPK/ERK1/2 (PD) and PI3-K/Akt (LY) in treatment of GBM^53–55^ and interestingly, the inhibitors of these pathways (PD and LY) in our study not only slightly reduced cell viability of U87MG cells but significantly exaggerated the cell damage induced by **7** and **4** after combined treatment (Table 3) which justify their addition as adjuvants for GBM treatment. In case of **6**, we did not find such an effect, which could point to possible involvement of inhibition of MAP/ERK1/2 or PI3-K/Akt pathways by this compound, an effect which should be experimentally verified in the future. It should be noted that dopamine receptors can activate various intracellular pathways like phosphatidylinositol 3-kinase/protein kinase Akt ( PI3K/Akt) or microtubule-associated protein kinases (MAPKs) like ERK1/2 and c-Jun N-terminal kinase (JNK) through receptor-dependent and independent mechanisms, though the specific pathways can vary depending on the receptor subtype and cell type^56–58^. Activation of D_1_-like dopamine receptors has been shown to stimulate PI3-K or ERK1/2^57,58^, whereas D_2_ and D_3_ receptors can also activate Akt through the Ras/ERK cascade^56,59^. However, a complex involving D_2_ receptors, β-arrestin 2, and Protein Phosphatase 2 A (PP2A) can dephosphorylate Akt, influencing its activity^60^. In case of possible involvement of the mTOR pathway, as studied with the inhibitor of this kinase, rapamycin (Rap), we demonstrated the cell-damaging effect of this compound, which supports previous findings in the GBM field^61^. For **6**, **7**, and **4**, we showed their synergistic cytotoxic action with this mTOR inhibitor (Table 3). Since Rap is also an autophagy inducer, it is not excluded that the investigated here hydantoin derivatives and Rap influence on this parameter to evoke higher cell damage when compared with their effects when given solely. However, this hypothesis also needs further experimental validation.

Another interesting observation from our study is evidence of a cell-damaging effect of two calpain inhibitors, MDL28170 and Calp, in U87MG cells, as well as their higher cytotoxic action with **6**, **7**, or **4** (Table 4). These findings support previous reports, which showed an involvement of calpains, calcium-dependent cysteine proteases, in the pathogenesis of various tumors, including GBM, and in drug resistance to various clinically used anticancer agents^62,63^. It has been shown that on the cellular level, pharmacological calpain inhibition increased the sensitivity of GBM cells towards TMZ^63^. In addition, a recent study demonstrated the involvement of calpain activation in angiogenesis mediated by GBM endothelial cells and pointed to calpain inhibitors as a potential therapeutic turning point^64^. Moreover, calpain inhibitors decreased GBM endothelial cells’ viability via inhibition of cell proliferation and apoptosis induction, and they synergized with TMZ in cytotoxic effects, suggesting the potential efficacy of a combined treatment^64^. Although the above findings highlight the potential of calpain inhibitor development as adjuvant drugs in GBM treatment, it should be noted that there are some studies showing the opposite data. For example, Cai et al. showed in U87MG and A172 cells that the activator of calpain, A23187 (25 µM), inhibited GBM cell growth as well as increased the TMZ-cytotoxic effect^65^. These effects were partially abolished by Calp, which, when given alone, increased GBM cell proliferation. In another study, the anticancer effects of D_1_R agonist, SKF83959, in U87MG cells were associated with mitochondrial injury, calpain activation, and endoplasmic reticulum stress, and these effects were reversed by calpain inhibitor, calpastatin^27^. Moreover, a study by Bassett et al. demonstrated that calpastatin phosphorylation promotes radiation resistance in GBM cells by increasing the activity of calpain proteases^66^. Interestingly, Masilamani et al. showed that calpain-mediated ZBTB18 cleavage product activates HIF1A-regulated genes, leading to increased lipid uptake, lipid droplets (LD) accumulation, and enhanced metabolic activity, increasing the tumorigenicity of GBM^67^. Also, in glioma stem cells (GSC), responsible for tumor recurrence, calpains could participate in the regulation of cell motility, which could be successfully inhibited by new experimental drug candidates^68^. Nevertheless, found in present study, the higher cytotoxic effect after combined treatment with compounds **6**, **7** and **4** and calpain inhibitors could be partially explained by the results of molecular docking to µ-calpain, in which we confirmed that the hydantoin moiety present in **6** and **7** might be a bioisoster of peptide bond(s) present in known calpain inhibitors (Figs. 9 and 10). Continuing future work on another derivative from the hydantoin-phenylpiperazine family, it will be crucial to validate these observations by measuring calpain expression and activity in GBM cells and/or testing in a cell-free system the impact of tested compounds on exogenously added calpain.

From a structural point of view, the most promising chemical combination for anticancer activity among the tested compounds is the N-butyl linker with benzhydryl moiety (compound **6**). Compared to compound **5**, the analogue without one phenyl moiety leads to the conclusion that the presence of two aromatic rings, closed but not directly bound to piperazine, might be necessary for the desired activity in GBM cells. For further studies, it might also be considered the introduction of a different bulky group instead of one of the phenyl rings. Interestingly, the most favourable substituents for cytotoxic activity (**6**) do not provide the best D_2_/D_4_R affinity (D_2_R K_i_ = 133 nM, D_4_R % inh = 30), whereas the compound **7** with relatively the highest D_2_/D_4_R affinity demonstrated lower than **6** anticancer activity (Tables 6 and 7). When compared the IC_50_ values of **6**, **7** and **4** between U87MG, A172 and U138MG and associating them with expression level D_2_R or D_4_R in these GBM cell lines (Table 7), we rather exclude the involvement of D_2_R or D_4_R in mechanism of anticancer activity of **6**, **7** or **4** which is also supported with analysis of their D_2_R or D_4_R affinity with cytotoxic activity. For example, compound **7**, which demonstrated the highest affinity to D_2_R and D_4_R, was not the best acting anticancer compound, and its cell-damaging effect differed between U87MG, A172, and U138MG cells regardless of the similar expression of these receptors in these cell lines. It should be noted that when compared of IC_50_ values from U87MG cells between our and Matteucci et al. study^24^, the best acting in our study 5-spirofluorenohydantoin derivatives (**6**, **7** and **4**) had better anti-glioblastoma activity (5, 33, 52 µM, respectively) when compared to the best acting compounds from arylpiperazines family **15** (IC_50_=80 µM) and **16** (IC_50_=118.3 µM), which possessed high affinity to D_2_R, D_3_R and D_4_R. However, further studies in a larger number of hydantoin-derived chemical families would be valuable for broad structure-activity relationship analysis and evaluation of the correlation between anticancer activity and dopaminergic or other target affinity.

The presented studies demonstrate valuable results, that fits in the scientific gap concerning both searching for efficient GBM therapeutics and evaluation of relevance of dopamine signalling in GBM progression. However, we are conscious of limitations, which will be included in our further work. Nevertheless, the usage of fibroblasts as a control cell line is a widely applied first choice; it is not an optimal solution, and in our future work, we will be testing the most active compounds also in human astrocytes^66,69^. In our study, we used GBM cell lines in a 2D cell culture system^27^. For further development, it will be crucial to expand the in vitro settings to patient-derived GBM cells and assays in 3D systems (e.g., spheroids, organoids) and finally, to confirm the efficacy in vivo using GBM xenograft models,^49,69,70^. Another issue is assessing the BBB penetrance of 5-spirohydantoin derivatives. Despite the very promising computational BBB predictions for **4**, **6**, and **7**, they should be further validated experimentally using PAMPA systems or multicellular in vitro BBB models^71,72^. In the study, we demonstrated a beneficial effect of a combination of 5-spirohydantoin derivatives with calpain inhibitors (Calp and MDL) or pro-survival intracellular pathways PI3-K/Akt or ERK1/2. Moreover, molecular docking predictions showed that compounds **4**, **6**, or **7** may directly modulate calpain activity. However, further experiments should be performed to explore and confirm these findings.

**Table 7:** The associative analysis between expression of D_2_ and D_4_ receptors was studied in three GBM cell lines, and the cytotoxic efficacy of **6**, **7**, and **4**.

	U87MG	A172	U138MG	SH-SY5Y
**D**_**2**_ **R**	+++	+++	+++	+
**D**_**4**_ **R**	++	++	++	+++
**6** IC_50_ in µM D_2_R Ki = 133 nM D_4_R inh. = 33%	15	5	27	n.s.
**7** IC_50_ in µM *D*_*2*_ *R Ki = 13 nM* *D*_*4*_ *R inh. = 95%*	33	21	57	n.s.
**4** IC_50_ in µM D_2_R Ki = 300 nM D_4_R inh. = 73%	52	56	> 1000	n.s.

D_2_R - dopamine receptor subtype 2; D_4_R - dopamine receptor subtype 4; n.s. – not studied.

## Conclusions

Taking into account the doubts concerning hypothesis that involvement of dopaminergic signalling pathway might play pivotal role in GBM growth and the appropriate antagonists could be an effective strategy to fight with this type of cancer, we decided to perform cytotoxic studies against GBM cells and non-tumour cells for our in-house series of eight 5-spirohydantoin derivatives with diversified D_2_R affinity (13 nM < *K*_*i*_ < 973 nM). Hence, we aimed to verify their relevance in both dopaminergic signalling and potential GBM treatment. Cytotoxic studies (the MTT and the LDH tests) with three GBM cell lines (U87MG, A172 and U138MG) and human skin fibroblasts (HSF) as non-tumor control, led us to select three of the most promising compounds (**4**, **6** and **7**) with selective anticancer activity much greater than that observed for clinically used chemotherapeutic TMZ. Moreover, we confirmed the potency of **4**, **6**, and **7** as adjuvant therapeutics based on the resulting their higher cell-damaging effect when combined with TMZ, even in the GBM-resistant phenotype (U138MG). Comparing the IC_50_ values between all three used GBM cell lines, dopamine receptors’ affinities, and associating this data with the expression level of D_2_-like receptors (DRs) in GBM cell lines, we rather exclude the involvement of these receptors in the cell-damaging mechanism of the investigated hydantoin derivatives. Our study confirms that it is relevant to evaluate towards cytotoxic activity all the series of compounds to exclude the potential coincidence of coexisting high DR’s affinity and anti-GBM activity. In our case, not the most potent DR’s ligands have the highest cell-damaging ability. Hence, further research leading to an explanation of the role of dopamine signaling in GBM growth is necessary. Interestingly, we observed a cell-damaging effect of calpain inhibitors in U87MG cells and their synergistic cytotoxic activity with **4**, **6** and **7**, which together with molecular docking studies, suggest that the tested hydantoins might also be calpain inhibitors. However, further studies, especially focusing on the measurement of calpain expression and activity, are needed.

## Supplementary Information

Below is the link to the electronic supplementary material.

Supplementary Material 1

Supplementary Material 2

## Data Availability

The datasets used and analyzed during the current study are available from the corresponding author on reasonable request.

## Abbreviations

5-HT_1A_: Serotonin receptor subtype 1A
5-HT_2A_: serotonin receptor subtype 2A
BBB: blood-brain barrier
BrdU: Bromo-2’-deoxyuridine
Calp: Calpeptin
D_2_R: Dopamine receptor subtype 2
D_3_R: Dopamine receptor subtype 3
D_4_R: Dopamine receptor subtype 4
FBS: Fetal bovine serum
Fer-1: Ferrostatin-1
GBM: Glioblastoma multiforme
HSF: Human skin fibroblast
LDH: Lactate dehydrogenase
MTT: 3-(4,5-dimethylthiazol-2-yl)-2,5-diphenyltetrazolium bromide
NAC: N-acetyl-cysteine
Nec-1: Necrostatin-1
PI: Propidium iodide
TMZ: Temozolomide

## References

[1] Rana, M., Liou, K. C., Thakur, A., Nepali, K. & Liou, J. P. Advancing glioblastoma therapy: Learning from the past and innovations for the future. *Cancer Lett.* **617**, 217601 (2025). 40037502 10.1016/j.canlet.2025.217601

[2] Sipos, D. et al. Glioblastoma: Clinical presentation, multidisciplinary management, and long-term outcomes. *Cancers (Basel)*. **17**, 146 (2025). 39796773 10.3390/cancers17010146 PMC11719842

[3] Le Calvez, K. et al. Adult glioblastoma in England: Incidence, treatment, and outcomes with novel population-based strata. *Cancer Epidemiol.* **97**, 102811 (2025). 40203511 10.1016/j.canep.2025.102811

[4] Stupp, R. et al. Radiotherapy plus concomitant and adjuvant temozolomide for glioblastoma. *N. Engl. J. Med.* **352**, 987–996 (2005). 15758009 10.1056/NEJMoa043330

[5] Lee, S. Y. Temozolomide resistance in glioblastoma multiforme. *Genes Dis.* **3**, 198–210 (2016). 30258889 10.1016/j.gendis.2016.04.007 PMC6150109

[6] Dong, Q., He, L., Chen, L. & Deng, Q. Opening the blood-brain barrier and improving the efficacy of temozolomide treatments of glioblastoma using pulsed, focused ultrasound with a microbubble contrast agent. Biomed Res Int. 6501508 (2018). 10.1155/2018/6501508 PMC6252217 30534564

[7] Li, Y., Ali, S., Clarke, J. & Cha, S. Bevacizumab in recurrent glioma: Patterns of treatment failure and implications. *Brain Tumor Res. Treat.* **5**, 1–9 (2017). 28516072 10.14791/btrt.2017.5.1.1 PMC5433944

[8] Liu, X. et al. Treatment mechanism and research progress of bevacizumab for glioblastoma. *Am. J. Cancer Res.* **15**, 1874–1901 (2025). 40371151 10.62347/RNUE7193 PMC12070100

[9] Xiao, Z. Z. et al. Carmustine as a supplementary therapeutic option for glioblastoma: A systematic review and meta-analysis. *Front. Neurol.* **11**, 1036 (2020). 33041980 10.3389/fneur.2020.01036 PMC7527463

[10] Herrlinger, U. et al. Lomustine-temozolomide combination therapy versus standard temozolomide therapy in patients with newly diagnosed glioblastoma with methylated MGMT promoter (CeTeG/NOA–09): A randomised, open-label, phase 3 trial. *Lancet* **393**, 678–688 (2019). 30782343 10.1016/S0140-6736(18)31791-4

[11] Prajapati, H. P. & Ansari, A. Updates in the management of recurrent glioblastoma multiforme. *J. Neurol. Surg. Cent. Eur. Neurosurg.* **84**, 174–187 (2023). 10.1055/s-0042-1749351 35772723

[12] Tang, X. et al. Targeting glioblastoma stem cells: A review on biomarkers, signal pathways and targeted therapy. *Front. Oncol.* **11**, 2703 (2021). 10.3389/fonc.2021.701291 PMC8297686 34307170

[13] Sharma, P., Aaroe, A., Liang, J. & Puduvalli, V. K. Tumor microenvironment in glioblastoma: Current and emerging concepts. *Neurooncol Adv.* **5**, vdad009 (2023). 36968288 10.1093/noajnl/vdad009 PMC10034917

[14] Rosas-Cruz, A., Salinas-Jazmín, N. & Velázquez, M. A. V. Dopamine receptors in cancer: Are they valid therapeutic targets? *Technol. Cancer Res. Treat.* **20**, 15330338211027913 (2021). 34212819 10.1177/15330338211027913 PMC8255587

[15] Abbruzzese, C., Matteoni, S., Persico, M., Villani, V. & Paggi, M. G. Repurposing chlorpromazine in the treatment of glioblastoma multiforme: Analysis of literature and forthcoming steps. *J. Experimental Clin. Cancer Res.* **39**, 26 (2020). 10.1186/s13046-020-1534-z PMC6995164 32005270

[16] Abbruzzese, C. et al. Drug repurposing for the treatment of glioblastoma multiforme. *J. Experimental Clin. Cancer Res.* **36**, 169 (2017). 10.1186/s13046-017-0642-x PMC5704391 29179732

[17] Tan, S. K. et al. Drug repositioning in glioblastoma: A pathway perspective. *Front. Pharmacol.* **9**, 218 (2018). 29615902 10.3389/fphar.2018.00218 PMC5864870

[18] Karpel-Massler, G. et al. Olanzapine inhibits proliferation, migration and anchorage-independent growth in human glioblastoma cell lines and enhances temozolomide’s antiproliferative effect. *J. Neurooncol*. **122**, 21–33 (2015). 25524815 10.1007/s11060-014-1688-7

[19] Weissenrieder, J. S., Neighbors, J. D., Mailman, R. B. & Hohl, R. J. Cancer and the dopamine D2 receptor: A pharmacological perspective. *J. Pharmacol. Exp. Ther.* **370**, 111–126 (2019). 31000578 10.1124/jpet.119.256818 PMC6558950

[20] Allen, J. E. et al. Discovery and clinical introduction of first-in-class imipridone ONC201. *Oncotarget*. **7**, 74380–74392 (2016). 10.18632/oncotarget.11814 PMC5342060 27602582

[21] Kline, C. L. B. et al. Role of Dopamine receptors in the anticancer activity of ONC201. *Neoplasia (United States)*. **20**, 80–91 (2018). 10.1016/j.neo.2017.10.002 PMC5725157 29216597

[22] Jeon, H. M. et al. Dopamine receptor D2 regulates glioblastoma survival and death through MET and death receptor 4/5. *Neoplasia* **39**, 100894 (2023). 36972629 10.1016/j.neo.2023.100894 PMC10066565

[23] Dolma, S. et al. Inhibition of dopamine receptor D4 impedes autophagic flux, proliferation, and survival of glioblastoma stem cells. *Cancer Cell.* **29**, 859–873 (2016). 27300435 10.1016/j.ccell.2016.05.002 PMC5968455

[24] Matteucci, F. et al. New arylpiperazines as potent and selective dopamine D4 receptor ligands potentially useful to treat glioblastoma. *J. Med. Chem.* **68**, 7441–7458 (2025). 40156554 10.1021/acs.jmedchem.4c03150

[25] Williford, S. E. et al. Novel dopamine receptor 3 antagonists inhibit the growth of primary and temozolomide resistant glioblastoma cells. *PLoS One*. **16**, e0250649 (2021). 33945569 10.1371/journal.pone.0250649 PMC8096095

[26] Kast, R. E., Ellingson, B. M., Marosi, C. & Halatsch, M. E. Glioblastoma treatment using perphenazine to block the subventricular zone’s tumor trophic functions. *J. Neurooncol*. **116**, 207–212 (2014). 24242756 10.1007/s11060-013-1308-y

[27] Yang, K., Xu, R. & Le, W. Dopamine receptor D1 agonist inhibits glioblastoma via calpain-mediated ER stress and mitochondrial dysfunction. *Oncol. Rep.* **45**, 74 (2021). 33760205 10.3892/or.2021.8025

[28] Xue, Z. et al. The dopamine receptor D1 inhibitor, SKF83566, suppresses GBM stemness and invasion through the DRD1-c-Myc-UHRF1 interactions. *J. Experimental Clin. Cancer Res.* **43**, 25 (2024). 10.1186/s13046-024-02947-7 PMC10801958 38246990

[29] Prabhu, V. V. et al. Dopamine receptor D5 is a modulator of tumor response to dopamine receptor D2 antagonism. *Clin. Cancer Res.* **25**, 2305–2313 (2019). 30559168 10.1158/1078-0432.CCR-18-2572 PMC7259201

[30] Karpel-Massler, G. et al. Anti-glioma activity of dapsone and its enhancement by synthetic chemical modification. *Neurochem Res.* **42**, 3382–3389 (2017). 28852934 10.1007/s11064-017-2378-6

[31] Roth, B. L., Tandra, S., Burgess, L. H., Sibley, D. R. & Meltzer, H. Y. D4 dopamine receptor binding affinity does not distinguish between typical and atypical antipsychotic drugs. *Psychopharmacol. (Berl)*. **120**, 365–368 (1995). 10.1007/BF02311185 8524985

[32] Matteucci, F. et al. Novel potent and selective dopamine D4 receptor piperidine antagonists as potential alternatives for the treatment of glioblastoma. *Pharmaceuticals* **18**, 739 (2025). 40430557 10.3390/ph18050739 PMC12114630

[33] Pavletić, P. et al. Highly potent and selective dopamine d4receptor antagonists potentially useful for the treatment of glioblastoma. *J. Med. Chem.* **65**, 12124–12139 (2022). 36098685 10.1021/acs.jmedchem.2c00840 PMC9511495

[34] Żesławska, E. et al. An insight into the structure of 5-spiro aromatic derivatives of imidazolidine-2,4-dione, a new group of very potent inhibitors of tumor multidrug resistance in T-lymphoma cells. *Bioorg. Chem.* **109**, 104735 (2021). 33640632 10.1016/j.bioorg.2021.104735

[35] Johnson, A. M. et al. Evaluation of histone deacetylase inhibitors as radiosensitizers for proton and light ion radiotherapy. *Front. Oncol.* **11**, 735940 (2021). 34513712 10.3389/fonc.2021.735940 PMC8426582

[36] Huang, H. M. et al. Enhancement of T2* Weighted MRI imaging sensitivity of U87MG glioblastoma cells using γ-ray irradiated low molecular weight hyaluronic acid-conjugated iron nanoparticles. *Int. J. Nanomed.* **16**, 3789–3802 (2021). 10.2147/IJN.S307648 PMC8179824 34103915

[37] Atif, F., Patel, N. R., Yousuf, S. & Stein, D. G. The synergistic effect of combination progesterone and temozolomide on human glioblastoma cells. *PLoS One*. **10**, e0131441 (2015). 26110872 10.1371/journal.pone.0131441 PMC4482510

[38] Lee, J. E., Yoon, S. S., Lee, J. W. & Moon, E. Y. Curcumin-induced cell death depends on the level of autophagic flux in A172 and U87MG human glioblastoma cells. *Chin. J. Nat. Med.* **18**, 114–122 (2020). 32172947 10.1016/S1875-5364(20)30012-1

[39] Matias, M., Silvestre, S., Falcão, A. & Alves, G. Considerations and Pitfalls in selecting the drug vehicles for evaluation of new drug candidates: Focus on in vivo pharmaco-toxicological assays based on the Rotarod performance test. *J. Pharm. Pharm. Sci.* **21**, 110–118 (2018). 29543586 10.18433/jpps29656

[40] Verheijen, M. et al. DMSO induces drastic changes in human cellular processes and epigenetic landscape in vitro. *Sci. Rep.* **9**, 4641 (2019). 30874586 10.1038/s41598-019-40660-0 PMC6420634

[41] Jantas, D. et al. An endogenous and ectopic expression of metabotropic glutamate receptor 8 (mGluR8) inhibits proliferation and increases chemosensitivity of human neuroblastoma and glioma cells. *Cancer Lett.* **432**, 1–16 (2018). 29885518 10.1016/j.canlet.2018.06.004

[42] Cheng, Y. & Prusoff, W. H. Relationship between the inhibition constant (K1) and the concentration of inhibitor which causes 50 per cent inhibition (I50) of an enzymatic reaction. *Biochem. Pharmacol.* **22**, 3099–3108 (1973). 4202581 10.1016/0006-2952(73)90196-2

[43] Van Tol, H. H. M. et al. Multiple dopamine D4 receptor variants in the human population. *Nature* **358**, 149–152 (1992). 1319557 10.1038/358149a0

[44] Weissenrieder, J. S. et al. Antipsychotic drugs elicit cytotoxicity in glioblastoma multiforme in a calcium-dependent, non-D2 receptor-dependent, manner. *Pharmacol. Res. Perspect.* **9**, e00689 (2021). 34003586 10.1002/prp2.689 PMC8130568

[45] Li, J. et al. Genome-wide shRNA screen revealed integrated mitogenic signaling between dopamine receptor D2 (DRD2) and epidermal growth factor receptor (EGFR) in glioblastoma. *Oncotarget* **5**, 882–893 (2014). 24658464 10.18632/oncotarget.1801 PMC4011590

[46] Xie, H. R., Hu, L., Sen & Li, G. Y. SH-SY5Y human neuroblastoma cell line: In vitro cell model of dopaminergic neurons in Parkinson’s disease. *Chin. Med. J. (Engl)*. **123**, 1086–1092 (2010). 20497720

[47] Donkor, I. O., Xu, J., Liu, J. & Cameron, K. Synthesis and antiproliferative activity of sulfonamide-based peptidomimetic calpain inhibitors. *Bioorg. Med. Chem.* **28**, 115433 (2020). 32199690 10.1016/j.bmc.2020.115433

[48] Jastaniah, A., Gaisina, I. N., Knopp, R. C. & Thatcher, G. R. J. Synthesis of α-ketoamide-based stereoselective Calpain-1 inhibitors as neuroprotective agents. *ChemMedChem* **15**, 2280–2285 (2020). 32840034 10.1002/cmdc.202000385 PMC8583780

[49] Garofano, L. et al. Pathway-based classification of glioblastoma uncovers a mitochondrial subtype with therapeutic vulnerabilities. *Nat. Cancer*. **2**, 141–156 (2021). 33681822 10.1038/s43018-020-00159-4 PMC7935068

[50] Moretti, I. F. et al. GBM cells exhibit susceptibility to metformin treatment according to TLR4 pathway activation and metabolic and antioxidant status. *Cancers (Basel)*. **15**, 587 (2023). 36765551 10.3390/cancers15030587 PMC9913744

[51] Galluzzi, L. & Kroemer, G. Secondary necrosis: Accidental no more. *Trends Cancer*. **3**, 1–2 (2017). 28718422 10.1016/j.trecan.2016.12.001

[52] Berghe, T. et al. Necroptosis, necrosis and secondary necrosis converge on similar cellular disintegration features. *Cell. Death Differ.* **17**, 922–930 (2010). 20010783 10.1038/cdd.2009.184

[53] McCubrey, J. A. et al. Roles of the RAF/MEK/ERK and PI3K/PTEN/AKT pathways in malignant transformation and drug resistance. *Adv. Enzyme Regul.* **46**, 249–279 (2006). 16854453 10.1016/j.advenzreg.2006.01.004

[54] Roskoski, R. The ErbB/HER family of protein-tyrosine kinases and cancer. *Pharmacol. Res.* **79**, 34–74 (2014). 24269963 10.1016/j.phrs.2013.11.002

[55] Sadremomtaz, A. et al. Dual blockade of VEGFR1 and VEGFR2 by a novel peptide abrogates VEGF-driven angiogenesis, tumor growth, and metastasis through PI3K/AKT and MAPK/ERK1/2 pathway. *Biochim. Biophys. Acta Gen. Subj.* **1862**, 2688–2700 (2018). 30251659 10.1016/j.bbagen.2018.08.013

[56] Welsh, G. I., Hall, D. A., Warnes, A., Strange, P. G. & Proud, C. G. Activation of microtubule-associated protein kinase (Erk) and p70 S6 kinase by D2 dopamine receptors. *J. Neurochem*. **70**, 2139–2146 (1998). 9572301 10.1046/j.1471-4159.1998.70052139.x

[57] Beaulieu, J. M. & Gainetdinov, R. R. The physiology, signaling, and pharmacology of dopamine receptors. *Pharmacol. Rev.* **63**, 182–217 (2011). 21303898 10.1124/pr.110.002642

[58] Spigolon, G., Cavaccini, A., Trusel, M., Tonini, R. & Fisone, G. cJun N-terminal kinase (JNK) mediates cortico-striatal signaling in a model of Parkinson’s disease. *Neurobiol. Dis.* **110**, 37–46 (2018). 29107639 10.1016/j.nbd.2017.10.015

[59] Brami-Cherrier, K. et al. Dopamine induces a PI3-kinase-independent activation of Akt in striatal neurons: a new route to cAMP response element-binding protein phosphorylation. *J. Neurosci.* **22**, 8911–8921 (2002). 12388598 10.1523/JNEUROSCI.22-20-08911.2002 PMC6757682

[60] Beaulieu, J. M., Espinoza, S. & Gainetdinov, R. R. Dopamine receptors - IUPHAR review 13. *Br. J. Pharmacol.* **172**, 1–23 (2015). 25671228 10.1111/bph.12906 PMC4280963

[61] Mecca, C., Giambanco, I., Donato, R. & Arcuri, C. Targeting mTOR in glioblastoma: Rationale and preclinical/clinical evidence. *Dis Markers* 9230479 (2018). (2018). 10.1155/2018/9230479 PMC6312595 30662577

[62] Storr, S. J., Carragher, N. O., Frame, M. C., Parr, T. & Martin, S. G. The calpain system and cancer. *Nat. Rev. Cancer*. **11**, 364–374 (2011). 21508973 10.1038/nrc3050

[63] Stillger, M. N. et al. Changes in calpain-2 expression during glioblastoma progression predisposes tumor cells to temozolomide resistance by minimizing DNA damage and p53-dependent apoptosis. *Cancer Cell Int.* **23**, 49 (2023). 36932402 10.1186/s12935-023-02889-8 PMC10022304

[64] Guarnaccia, L. et al. Testing calpain inhibition in tumor endothelial cells: novel targetable biomarkers against glioblastoma malignancy. *Front. Oncol.* **14**, 1355202 (2024). 39156707 10.3389/fonc.2024.1355202 PMC11327812

[65] Cai, L. et al. Calpain suppresses cell growth and invasion of glioblastoma multiforme by producing the cleavage of Filamin A. *Int. J. Clin. Oncol.* **25**, 1055–1066 (2020). 32103382 10.1007/s10147-020-01636-7

[66] Bassett, E. A. et al. Calpastatin phosphorylation regulates radiation-induced calpain activity in glioblastoma. *Oncotarget* **9**, 14597–14607 (2018). 29581866 10.18632/oncotarget.24523 PMC5865692

[67] Masilamani, A. P. et al. Calpain-mediated cleavage generates a ZBTB18 N-terminal product that regulates HIF1A signaling and glioblastoma metabolism. *iScience* **25**, 104625 (2022). 35800763 10.1016/j.isci.2022.104625 PMC9253709

[68] Marín-Ramos, N. I. et al. Inhibition of motility by NEO100 through the calpain-1/RhoA pathway. *J. Neurosurg.* **133**, 1020–1031 (2019). 31419797 10.3171/2019.5.JNS19798

[69] Sivakumar, H., Devarasetty, M., Kram, D. E., Strowd, R. E. & Skardal, A. Multi-cell type glioblastoma tumor spheroids for evaluating sub-population-specific drug response. *Front. Bioeng. Biotechnol.* **8**, 538663 (2020). 33042963 10.3389/fbioe.2020.538663 PMC7523412

[70] Jeon, H. M. et al. Dopamine receptor D2 regulates glioblastoma survival and death through MET and death receptor 4/5. *Neoplasia* **39**, 100894 (2023). 36972629 10.1016/j.neo.2023.100894 PMC10066565

[71] Digiovanni, S. et al. Blood-brain barrier permeability increases with the differentiation of glioblastoma cells in vitro. *Fluids Barriers CNS*. **21**, 89 (2024). 39487455 10.1186/s12987-024-00590-0 PMC11529439

[72] Dyshlovoy, S. A. et al. Rhizochalinin exhibits anticancer activity and synergizes with EGFR inhibitors in glioblastoma in vitro models. *Mol. Pharm.* **20**, 4994–5005 (2023). 37733943 10.1021/acs.molpharmaceut.3c00217

