# Supplementary material for: Spirohydantoin derivatives exert dopamine D2-like receptor-independent cytotoxicity in glioblastoma cells with possible involvement of calpain inhibition
Source: Sci Rep. 2026 Mar 24;16:14883. doi: 10.1038/s41598-026-43014-9 (PMC13168489; doi:10.1038/s41598-026-43014-9)
Supplement: Supplementary file 1 — Supplementary Material 1 [file 41598_2026_43014_MOESM1_ESM.pdf]

Supplementary materials – Part 1

Spirohydantoin derivatives exert dopamine D_2-like receptor-independent cytotoxicity in glioblastoma cells with possible involvement of calpain inhibition

Katarzyna Kucwaj-Brysz[1*], Sabina Podlewska[2], Klaudia Jakubowska[2], Aleksandra Mąsior[2], Michał Wilczkowski[2], Beata Duszyńska[2], Justyna Drukała[3], Jadwiga Handzlik[1] and Danuta Jantas[2*]

[1]*Chair of Chemical Technology and Biotechnology of Drugs, Jagiellonian University Medical College, Medyczna 9, 30-688, Cracow, Poland*

[2]*Maj Institute of Pharmacology, Polish Academy of Sciences, Smętna 12, 31-343, Cracow, Poland*

[4]*Department of Cell Biology, Faculty of Biochemistry, Biophysics and Biotechnology, Jagiellonian University, Gronostajowa 7, 30-387, Cracow, Poland*

*****correspondence: katarzyna.kucwaj@uj.edu.pl (KK-B)and jantas@if-pan.krakow.pl (DJ)

Table S1. Chemical structure of hydantoin-phenylpiperazines compounds **1–8**.

**Compound**	**n**	**R**
**1**	1	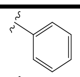
**2**	2	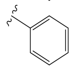
**3**	2	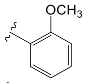
**4**	2	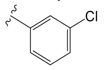
**5**	2	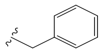
**6**	2	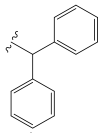
**7**	3	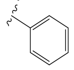
**8**	3	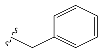

n – number of methylene group(s) in linker; R – aromatic substituent

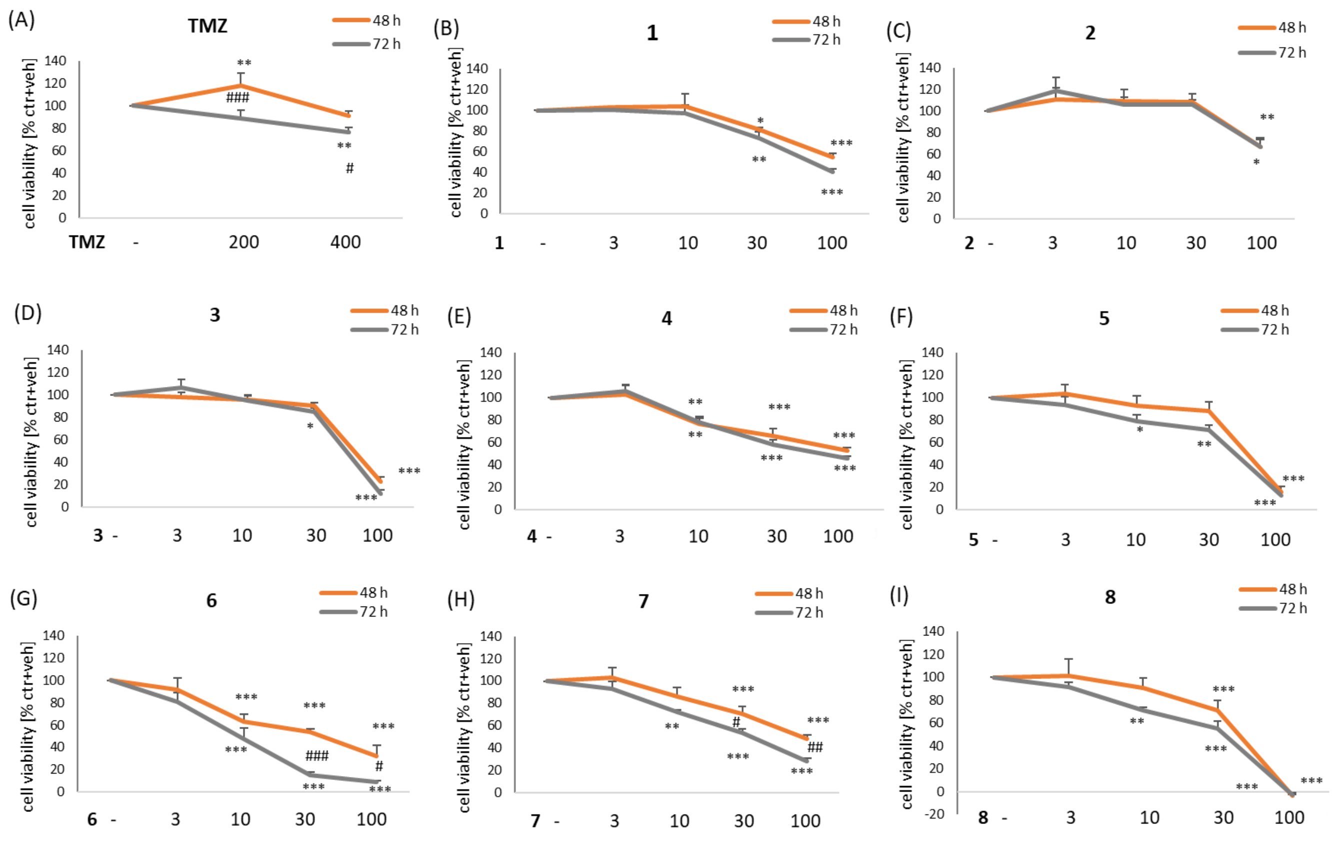

Figure S1. The cytotoxic effect of temozolomide (TMZ) and compounds **1-8** in U87MG cells cultured in medium without FBS. The cells were treated with TMZ (400 and 800 μM) or **1-8** compounds (at concentrations 3, 10, 30 and 100 μM) for 48 and 72 h. Cell viability was estimated by MTT reduction test. The data were normalized to vehicle-treated cells and are presented as mean ± SEM from 4 independent experiments. $^{*}P<0.05$, $^{**}P<0.01$and $^{***}P<0.001$ vs. vehicle-treated cells; $^{\#}P<0.05$, $^{\#\#}P<0.01$ and $^{\#\#\#}P<0.001$ vs. 48 h.

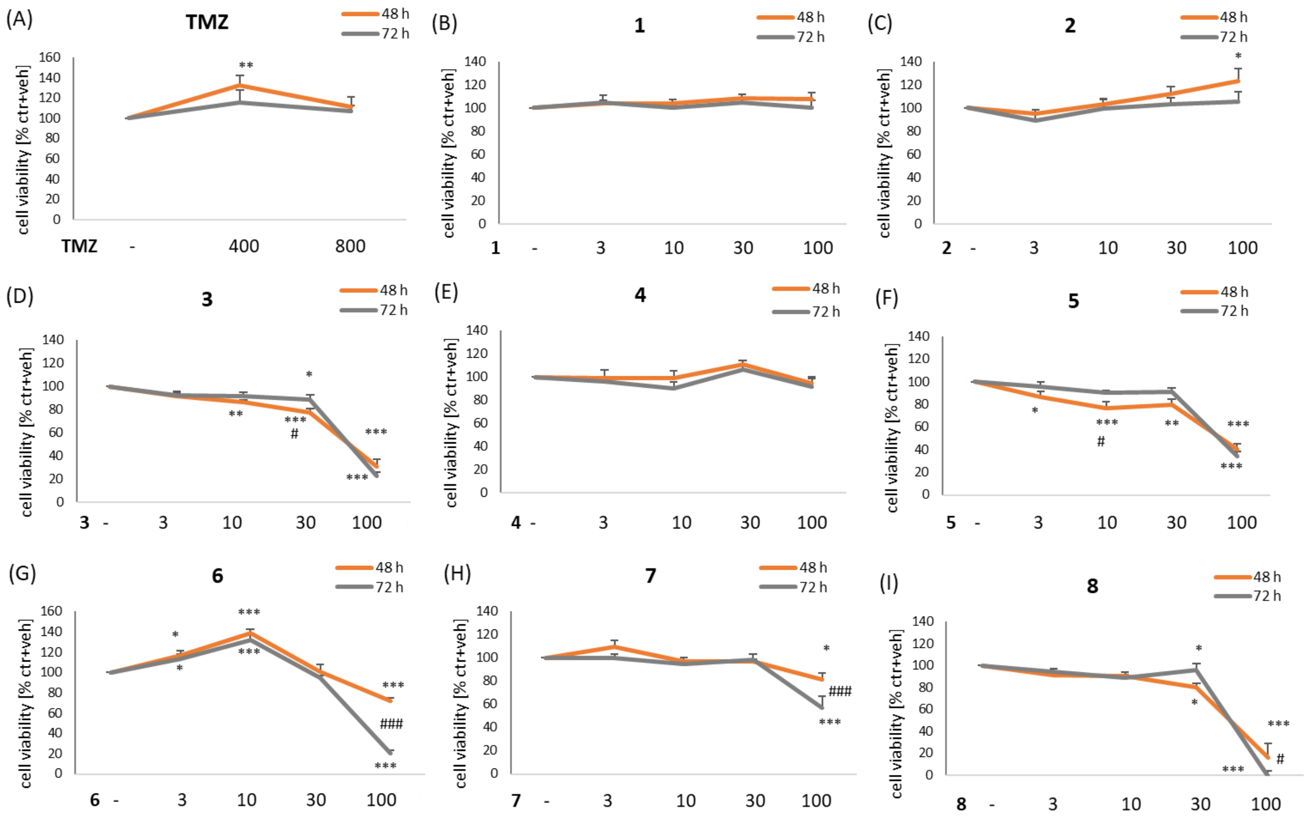

Figure S2. The effects of temozolomide (TMZ) and compounds **1-8** in human skin fibroblasts (HSF). The cells were treated with TMZ (400 and 800 μM) or **1-8** compounds (at concentrations 3, 10, 30 and 100 μM) for 48 and 72 h. Cell viability was estimated by MTT reduction test. The data were normalized to vehicle-treated cells and are presented as mean ± SEM from 5 independent experiments. *P<0.05, **P<0.01and ***P<0.001 vs. vehicle-treated cells; $^{\#}$P<0.05 and $^{\#\#\#}$P<0.001 vs. 48 h.

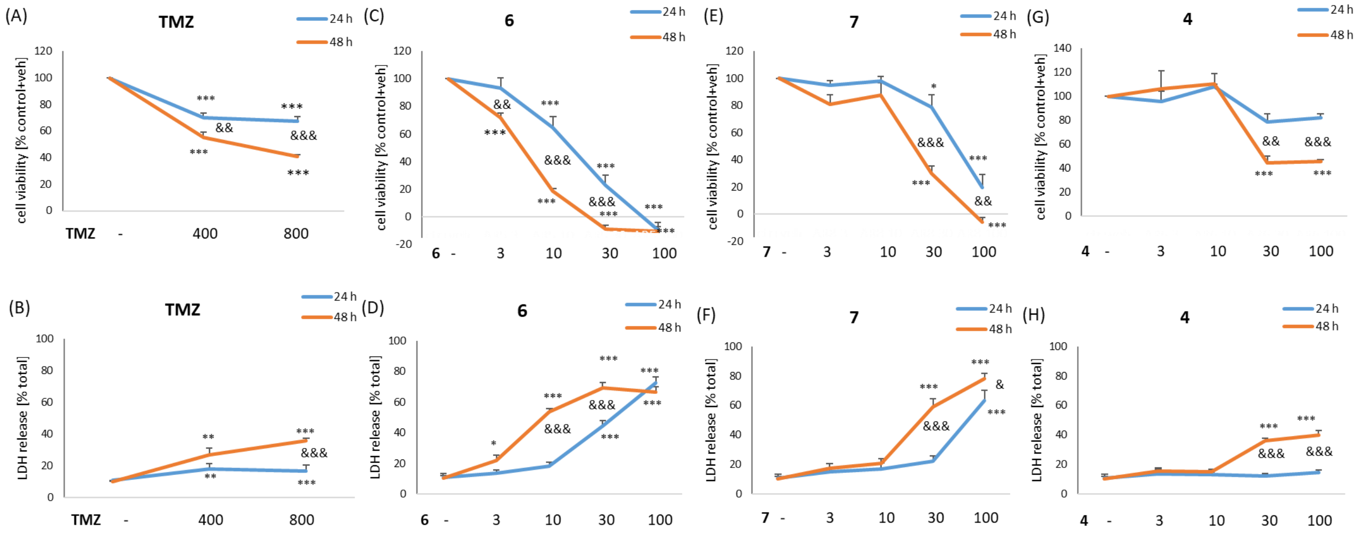

Figure S3. The effects of temozolomide (TMZ) and compounds **6, 7** and **4** in A172 cells. The cells were treated with TMZ (400 and 800 μM) or compounds **6, 7** or **4** (at concentrations 3, 10, 30 and 100 μM) for 24 and 48 h. Cell viability (A,C,E,G) and cytotoxicity (B,D,F,I) were estimated by MTT reduction and LDH release tests, respectively. The data were normalized to vehicle-treated cells and are presented as mean ± SEM from 4 independent experiments. *P<0.05, **P<0.01 and ***P<0.001 vs. vehicle-treated cells; $^{\&}$P<0.05, $^{\&\&}$P<0.01 and $^{\&\&\&}$P<0.001 vs. 24 h.

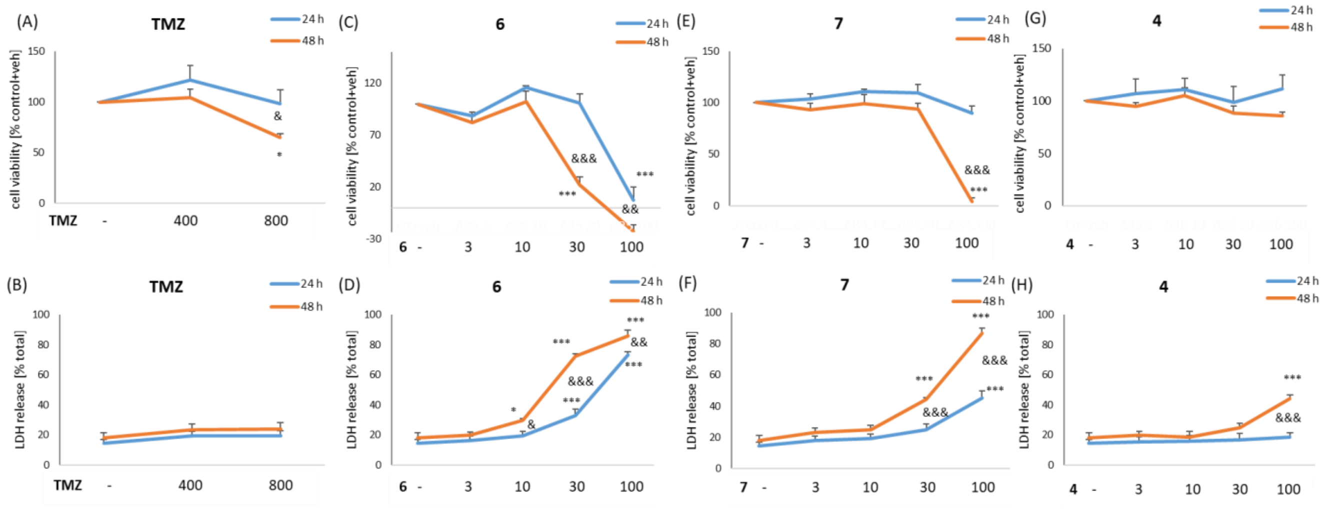

Figure S4. The effects of temozolomide (TMZ) and compounds **6**, **7** and **4** in U138MG cells. The cells were treated with TMZ (400 and 800 μM) or **6, 7** or **4** (at concentrations 3, 10, 30 and 100 μM) for 24 and 48 h. Cell viability (A,C,E,G) and cytotoxicity (B,D,F,I) were estimated by MTT reduction and LDH release tests, respectively. The data were normalized to vehicle-treated cells and are presented as mean ± SEM from 4 independent experiments. *P<0.05, **P<0.01and ***P<0.001 vs. vehicle-treated cells; $^{\&}$P<0.05, $^{\&\&}$P<0.01 and $^{\&\&\&}$P<0.001 vs. 24 h.

Table S2. The effect of antioxidant and inhibitors of apoptosis, necroptosis and ferroptosis on cell damage induced by compounds **6**, **7** and **4** in U87MG cells.

	**24 h**	**48 h**
control+veh	100.00 ± 0.00	100.00 ± 0.00
**6**	41.73 ± 2.54 ***	23.19 ± 1.84 ***
**6** + NAC	43.31 ± 4.67 ***	24.48 ± 0.51 ***
**6** + Ac	35.72 ± 3.79 ***	23.58 ± 2.95 ***
**6** + Nec-1	41.86 ± 3.03 ***	26.79 ± 0.82 ***
**6** + Fer-1	44.84 ± 2.35 ***	27.01 ± 1.97 ***
**7**	70.13 ± 4.99 ***	69.30 ± 4.67 ***
**7** + NAC	76.07 ± 6.26 ***	74.02 ± 6.34 ***
**7** + Ac	60.32 ± 0.17 ***	61.90 ± 4.30 ***
**7** + Nec-1	64.24 ± 2.92 ***	64.01 ± 5.11 ***
**7** + Fer-1	71.29 ± 3.10 ***	66.19 ± 1.42 ***
**4**	59.86 ± 3.40 ***	59.48 ± 4.50 ***
**4** + NAC	66.52 ± 2.69 ***	61.47 ± 12.76 ***
**4** + Ac	57.61 ± 3.44 ***	55.12 ± 4.14 ***
**4** + Nec-1	58.26 ± 6.28 ***	56.85 ± 5.18 ***
**4** + Fer-1	66.05 ± 5.89 ***	60.52 ± 3.80 ***
Ac + veh	75.66 ± 1.85 ***	89.16 ± 1.00
Nec-1 + veh	95.02 ± 9.75	98.20 ± 9.24
Fer-1 + veh	91.39 ± 4.22	96.70 ± 1.25

The U87MG cells were pre-treated for 30 min with an antioxidant - N-acetyl-cysteine (NAC, 1 mM), caspase-3 inhibitor - Ac-DEVD-CHO (Ac, 20 μM), necroptosis inhibitor - necrostatin-1 (Nec-1, 20 μM) or ferroptosis inhibitor - ferrostatin-1 (Fer-1, 20 μM) followed by 24 or 48 h exposure to 30 μM of **6**, **7** and **4**. Cell viability was estimated by MTT reduction test. The data were normalized to vehicle-treated cells (H_2O/DMSO) and are presented as mean ± SEM from 3-8 independent experiments. ***P<0.001 vs. vehicle-treated cells.

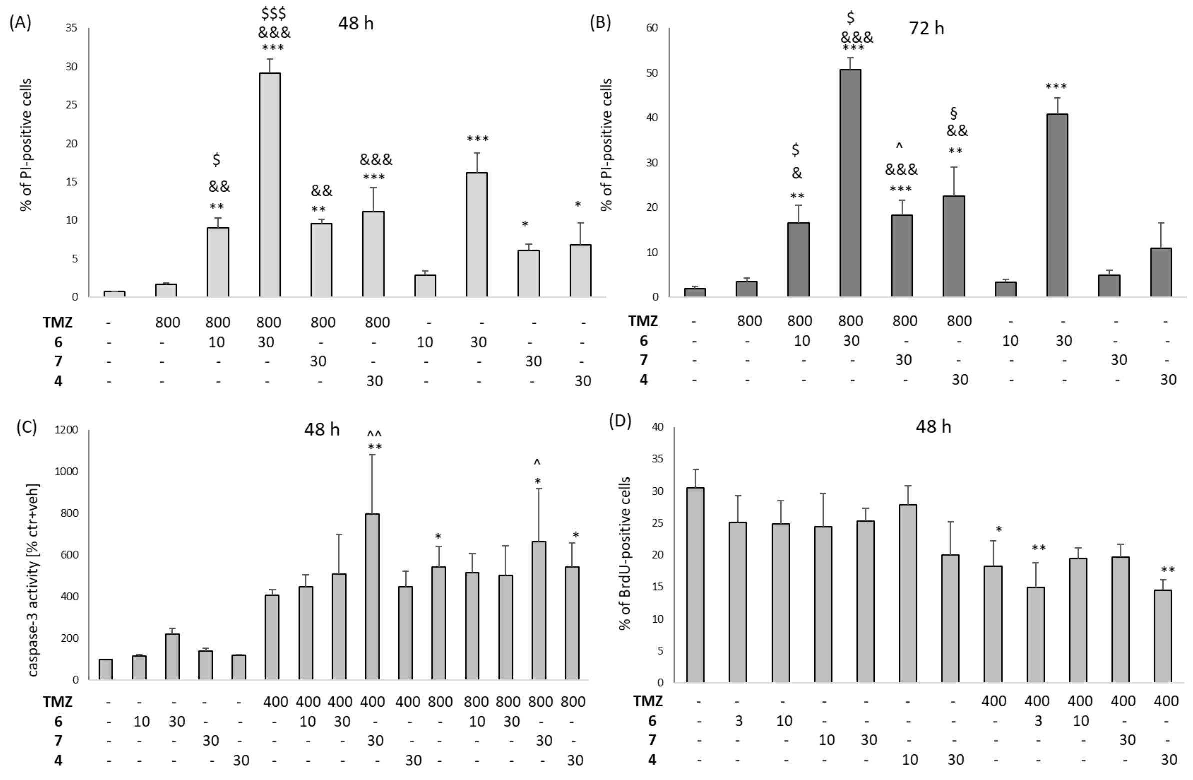

Fig. S5. The effects of combinatory treatment of TMZ with compounds **6**, **4** and **7** on necrotic (A, B), apoptotic (C) and cell proliferation (D) markers. The cells were treated with TMZ (400 or 800 μM) or compounds **6** (3-30 μM), **4** (30 μM) or **7** (3-30 μM) for 48-72 h (necrosis) and 24-48 h (apoptosis). Necrotic changes were estimated by propidium iodide (PI) staining (A), apoptotic ones by caspase-3 activity measurement (B) and cell proliferation by BrdU staining. The data are presented as mean ± SEM of % of PI positive cells (A, B), % of vehicle-treated cells for caspase-3 activity (C) and % of BrdU-positive cells (D) from 3-4 independent experiments. *P<0.05, **P<0.01 and ***P<0.001 vs. vehicle-treated cells; $^{\&}$P<0.05, $^{\&\&}$P<0.01 and $^{\&\&\&}$P<0.001 vs. TMZ; $^{\$}$P<0.05, $^{\$\$}$P<0.01 and $^{\$\$\$}$P<0.001vs. **6** at particular concentration; $^{\wedge}$P<0.05 and $^{\wedge\wedge}$P<0.01 vs. **7;** §P<0.05 vs. **4**.

Table S3. The effect of combined treatment of **6**, **7** or **4** with TMZ in A172 cells.

	24 h		48 h	
	MTT test	*LDH test*	*MTT test*	*LDH test*
control+veh	100.00 ± 0.00	10.97 ± 2.41	100.00 ± 0.00	10.34 ± 1.58
**6**	64.63 ± 8.10 **	18.28 ± 2.47	18.78 ± 1.50 ***	53.41 ± 2.50 ***
**7**	78.67 ± 9.33 *	22.44 ± 3.54 **	30.04 ± 5.57 ***	58.99 ± 5.36 ***
**6**	78.66 ± 6.64 *	12.04 ± 1.88	44.60 ± 5.32 ***	35.91 ± 5.36 ***
TMZ	70.00 ± 3.28 *	18.31 ± 2.01 *	55.09 ± 4.19 ***	26.96 ± 4.63 **
**6** + TMZ	46.06 ± 8.17 $^{***,\#}$	25.60 ± 1.56 **	2.76 ± 2.83 $^{***,\#\#\#,\$\$}$	53.45 ± 1.31 $^{***,\#\#\#}$
**7** + TMZ	44.82 ± 6.00 $^{***,\#,\wedge}$	36.81 ± 3.08 $^{***,\#\#\#,\wedge\wedge\wedge}$	0.00 ± 5.41 $^{***,\#\#\#,\wedge\wedge\wedge}$	67.74 ± 3.16 $^{***,\#\#\#}$
**4** + TMZ	56.86 ± 6.01 ***,§	21.97 ± 2.51 *,§	2.49 ± 3.05 $^{***,\#\#\#,\S\S\S}$	71.33 ± 2.88 $^{***,\#\#\#,\S\S\S}$

The A172 cells were treated with **6** (10 μM), **7** (30 μM) or **4** (30 μM) alone or in combination with TMZ (400 μM). Cell viability and toxicity were estimated by MTT reduction and LDH release assays, respectively. The data were normalized to vehicle-treated cells (MTT test) or $TritonX_{100}$-treated cells (LDH test) and are presented as mean ± SEM from 4 independent experiments. *P<0.05, **P<0.01 and ***P<0.001 vs. vehicle-treated cells; $^{\#}$P<0.05 and $^{\#\#\#}$P<0.001 vs. TMZ-treated cells; $^{\$\$}$P<0.01 vs. **6**-treated cells; $^{\wedge}$P<0.05 and $^{\wedge\wedge\wedge}$P<0.001 vs. **7**-treated cells; §P<0.05 and §§§P<0.001 vs. **4**-treated cells.

Table S4. The effect of combined treatment of **6**, **7** or **4** with TMZ in U138MG cells.

	24 h		48 h	
	MTT test	*LDH test*	*MTT test*	*LDH test*
control+veh	100.00 ± 0.00	14.42 ± 2.64	100.00 ± 0.00	18.08 ± 3.12
**6** 10	115.69 ± 1.84	19.41 ± 2.92	102.03 ± 10.62	29.80 ± 1.27 **
**6** 30	100.89 ± 8.75	32.79 ± 4.47 **	30.11 ± 2.24 ***	72.37 ± 1.49 ***
**7**	109.52 ± 8.42	24.77 ± 3.90	93.86 ± 6.37	44.19 ± 1.43 ***
**4**	98.68 ± 15.06	16.92 ± 3.99	88.28 ± 6.59	24.85 ± 2.84
TMZ	121.87 ± 14.05	19.34 ± 3.17	104.48 ± 8.29	23.26 ± 3.87
**6** 10 + TMZ	97.13 ± 8.88	21.37 ± 3.06	81.32 ± 8.01 $^{\#}$	31.01 ± 3.04 **
**6** 30 + TMZ	95.04 ± 13.41	35.43 ± 2.94 $^{***,\#\#}$	19.22 ± 4.19 $^{***,\#\#\#}$	70.06 ± 2.67 $^{***,\#\#}$
**7** + TMZ	85.31 ± 4.51 $^{\#}$	24.85 ± 3.58	70.77 ± 6.09 $^{**,\#\#,\wedge}$	34.19 ± 1.49 $^{***,\#,\wedge\wedge}$
**4** + TMZ	86.74 ± 10.75 $^{\#}$	23.07 ± 3.14	75.94 ± 3.82 $^{*,\#}$	30.87 ± 2.90 **

The U138MG cells were treated with 6 (10 and 30 μM), 7 (30 μM) or 4 (30 μM) alone or in combination with TMZ (400 μM). Cell viability and toxicity were estimated by MTT reduction and LDH release assays, respectively. The data were normalized to vehicle-treated cells (MTT test) or TritonX$_{100}$-treated cells (LDH test) and are presented as mean ± SEM from 4 independent experiments. *P<0.05, **P<0.01 and ***P<0.001 vs. vehicle-treated cells; $^{\#}$P<0.05, $^{\#\#}$P<0.05 and $^{\#\#\#}$P<0.001 vs. TMZ-treated cells; $^{\wedge}$P<0.05 and $^{\wedge\wedge}$P<0.01 vs. **7**-treated cells.

Fig. S6. Ligand-protein interaction patterns evolution in time during MD simulations carried out for compounds **6**, **7** and calpeptin.

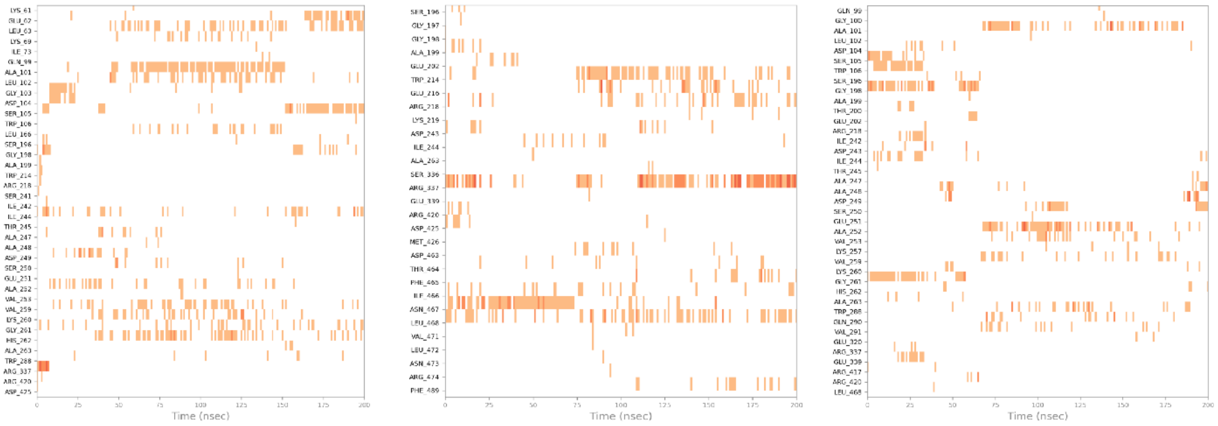

6 **7** **calpeptin**

Fig. S7. Analysis of different energy components calculated via MM-GBSA approach.

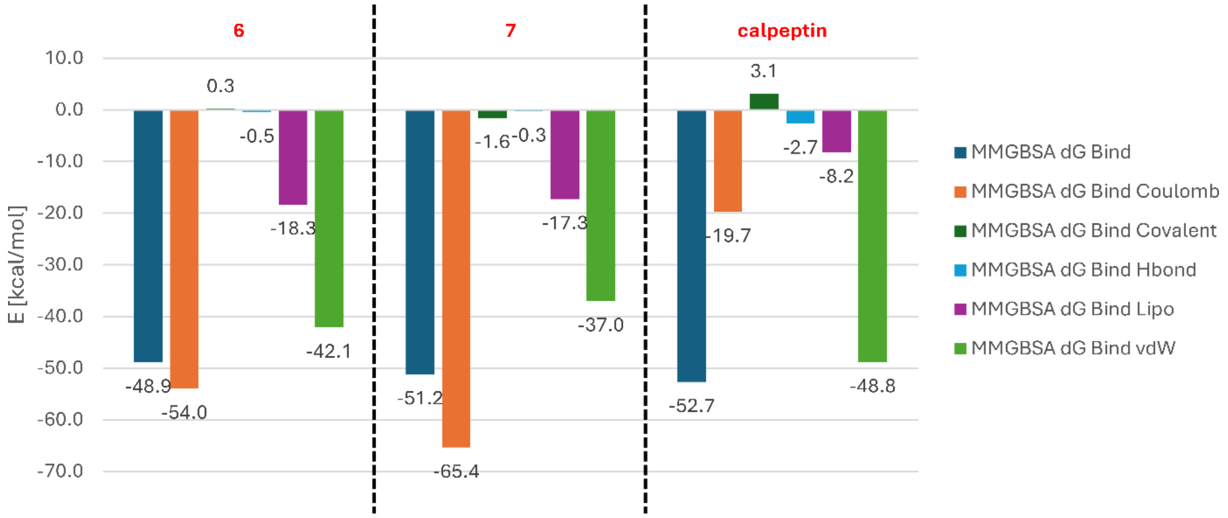

Fig. S8. Analysis of radius of gyration obtained during MD simulations studies for compounds **6**, **7** and calpeptin

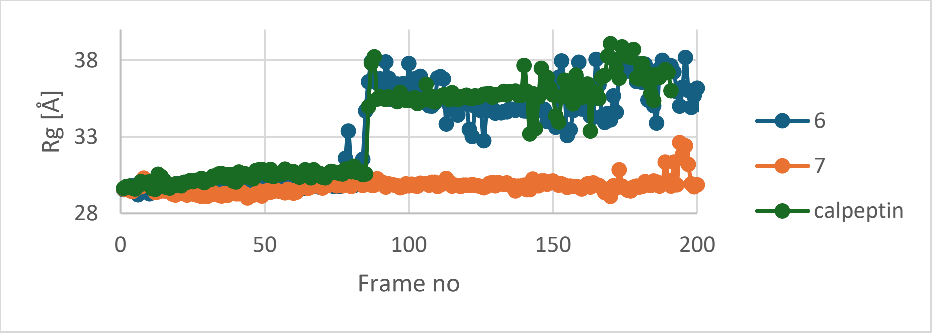

Fig. S9. Analysis of solvent-accessible surface area (SASA)obtained during MD simulations studies for compounds **6**, **7** and calpeptin

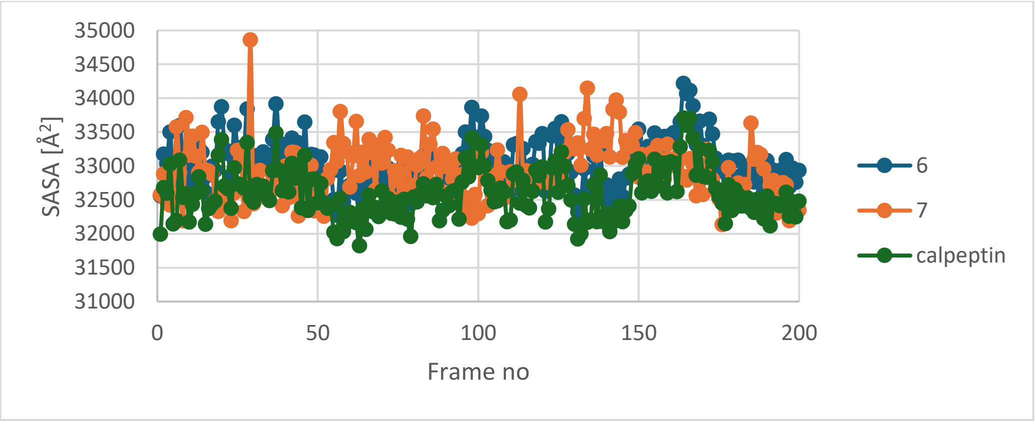

Results of ANOVA tests performed for the study

Results of two-way ANOVA tests for Figure 4
Figure 3A: F(4, 136)=1.4553 p=0.21929
Figure 3B: F(8, 60)=2.3724 p=0.02727
Figure 3C: F(8, 60)=1.5317 p=0.16548
Figure 3D: F(8, 60)=3.2139 p=0.00419
Figure 3E: F(8, 60)=2.5644 p=0.01781
Figure 3F: F(8, 59)=5.2438 p=0.00006
Figure 3G: F(8, 60)=4.4866 p=0.00026
Figure 3H: F(8, 60)=7.2431 p=0.00000
Figure 3I: F(8, 60)=1.3646 p=0.23056

Results of two-way ANOVA tests for Figure S1
Figure S1A: F(2, 34)=4.6837 p=0.01597
Figure S1B: F(4, 30)=0.52176 p=0.72042
Figure S1C: F(4, 30)=0.15643 p=0.95857
Figure S1D: F(4, 30)=1.5754 p=0.20640
Figure S1E: F(4, 30)=0.50553 p=0.73195
Figure S1F: F(4, 30)=0.62966 p=0.64513
Figure S1G: F(4, 30)=2.3977 p=0.07225
Figure S1H: F(4, 30)=1.1997 p=0.33133
Figure S1I: F(4, 30)=0.87921 p=0.48805

Results of two-way ANOVA tests for Figure S2
Figure S2A: F(2, 24)=1.0999 p=0.3491
Figure S2B: F(4, 40)=0.3577 p=0.83848
Figure S2C: F(4, 40)=0.56122 p=0.69211
Figure S2D: F(4, 40)=2.4793 p=0.05928
Figure S2E: F(4, 40)=0.19222 p=0.94106
Figure S2F: F(4, 40)=2.3581 p=0.06977
Figure S2G: F(4, 40)=13.300 p=0.0000
Figure S2H: F(4, 40)=2.4216 p=0.06406
Figure S2I: F(4, 40)=2.4038 p=0.06561

**Results of two-way ANOVA tests for Figure S3**
Figure S3A: F(2, 18)=12.079 p=0.00047
Figure S3B: F(2, 18)=5.5109 p=0.01358
Figure S3C: F(4, 30)=8.3394 p=0.00012
Figure S3D: F(4, 30)=18.170 p=0.00000
Figure S3E: F(4, 30)=4.6552 p=0.0482
Figure S3F: F(4, 30)=7.8880 p=0.00018
Figure S3G: F(4, 30)=5.4142 p=0.00210
Figure S3H: F(4, 30)=17.360 p=0.00000

**Results of two-way ANOVA tests for Figure S4**
Figure S4A: F(2, 18)=1.7109 p=0.20880
Figure S4B: F(2, 18)=0.00615 p=0.99387
Figure S4C: F(4, 30)=9.8984 p=0.00003
Figure S4D: F(4, 30)=13.773 p=0.00000
Figure S4E: F(4, 30)=20.105 p=0.00000
Figure S4F: F(4, 30)=13.769 p=0.00000
Figure S4G: F(4, 30)=0.54826 p=0.70167
Figure S4H: F(4, 30)=4.5736 p=0.00529

**Results of two-way ANOVA tests for Figure 5**
Figure 4A: F(10, 92)=25.313 p=0.0000
Figure 4B: F(10, 67)=10.254 p=0.00000

**Results of one-way ANOVA tests for Table S1**
for 24 h analysis: F(18, 52)=18.328 p=0.00000
for 48 h analysis: F(18, 59)=31.285 p=0.0000

**Results of one-way ANOVA tests for Table 4**
for 24 h analysis: F(23, 73)=33.067 p=0.0000
for 48 h analysis: F(23, 74)=36.510 p=0.0000

**Results of one-way ANOVA tests for Table 5**
for 24 h analysis: F(15, 63)=48.657 p=0.0000
for 48 h analysis: F(15, 66)=38.710 p=0.0000

Results of one-way ANOVA tests for Figure 6
A: F(17, 88)=17.470 p=0.0000
B: F(17, 36)=13.105 p=0.0000
C: F(17, 86)=48.385 p=0.0000
D: F(17, 36)=10.496 p=0.00000

Results of one-way ANOVA tests for Figure S5
A: F(9, 70)=23.318 p=0.0000
B: F(9, 69)=22.800 p=0.0000
C: F(14, 30)=2.9035 p=0.00698
D: F(11, 60)=2.0906 p=0.03482

Results of one-way ANOVA tests for Table S2
MTT test 24 h: F(7, 23)=8.0050 p=0.00006
LDH test for 24 h: F(7, 23)=10.348 p=0.00001
MTT test 48 h: F(7, 24)=74.919 p=0.0000
LDH test for 48 h: F(7, 24)=43.144 p=0.00000

Results of one-way ANOVA tests for Table S3
MTT test 24 h: F(9, 30)=1.4104 p=0.22774
LDH test for 24 h: F(9, 30)=3.7476 p=0.00297
MTT test 48 h: F(9, 29)=19.694 p=0.00000
LDH test for 48 h: F(9, 30)=54.397 p=0.00000

Results of one- or two-way ANOVA tests for Figure 8
A one-way: F(3, 18)=34.366 p=0.00000
B one-way: F(3, 18)=2.2382 p=0.11877
D two-way: F(3, 36)=2.7796 p=0.05501
