# Supplementary material for: Spirohydantoin derivatives exert dopamine D2-like receptor-independent cytotoxicity in glioblastoma cells with possible involvement of calpain inhibition
Source: Sci Rep. 2026 Mar 24;16:14883. doi: 10.1038/s41598-026-43014-9 (PMC13168489; doi:10.1038/s41598-026-43014-9)
Supplement: Supplementary file 2 — Supplementary Material 2 [file 41598_2026_43014_MOESM2_ESM.pdf]

Supplementary materials – Part 2

Spirohydantoin derivatives exert dopamine D2-like receptor-independent cytotoxicity in glioblastoma cells with possible involvement of calpain inhibition

Katarzyna Kucwaj-Brysz[1*], Sabina Podlewska[2], Klaudia Jakubowska[2], Aleksandra Mąsior[2], Michał Wilczkowski[2], Beata Duszyńska[2], Justyna Drukała[3], Jadwiga Handzlik[1] and Danuta Jantas[2*]

[1]*Chair of Chemical Technology and Biotechnology of Drugs, Jagiellonian University Medical College, Medyczna 9, 30-688, Cracow, Poland*

[2]*Maj Institute of Pharmacology, Polish Academy of Sciences, Smętna 12, 31-343, Cracow, Poland*

[4]*Department of Cell Biology, Faculty of Biochemistry, Biophysics and Biotechnology, Jagiellonian University, Gronostajowa 7, 30-387, Cracow, Poland*

***correspondence:** *katarzyna.kucwaj@uj.edu.pl (KK-B)and jantas@if-pan.krakow.pl (DJ)*

The file contain original data from expression of D_2-like receptors (D_2R, D_3R and D_4R) as well total protein measurements in the GBM cell lines (U87MG, A172 and U138MG). The human neuroblastoma SH-SY5Y cells (undifferentiated and differentiated phenotype) were used as a reference human cell line with dopaminergic phenotype. The measurments were done with ProteinSimple Jess, an automated capillary-based western blot platform (Bio-Techne, Minneapolis, MN, USA). The data are from two independent experiments with three repetition in each.

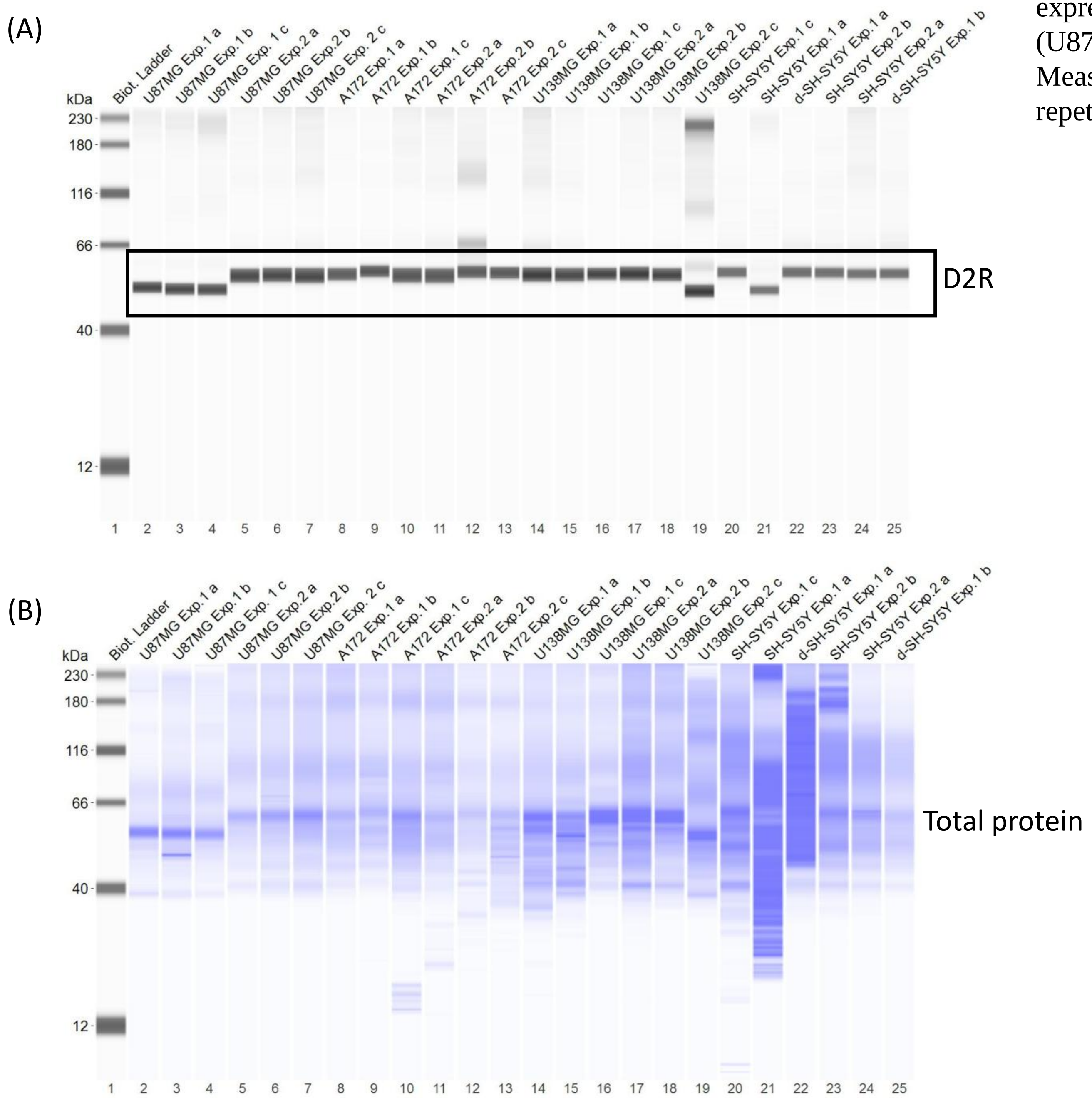

Fig. SII.1. Portrayal of a traditional blot-like image with a lane view of D_2 receptor expression (panel A) and total protein (panel B) of human glioblastoma cell lines (U87MG, A172, U138MG) and human neuroblastoma SH-SY5Y cells probes. Measurments were done on probes from two independent experiments with three repetitions in each (a, b, c).

Probes description and MW of D_2R from electropherogram for each probe (52-58 kDa) - 25 probes microcapillary system

1) Biot. Ladder
2) U87MG Exp. 1a, MW = 53
3) U87MG Exp. 1b, MW = 53
4) U87MG Exp. 1c, MW = 52
5) U87MG Exp. 2a, MW = 57
6) U87MG Exp. 2b, MW = 57
7) U87MG Exp. 2c, MW = 57
8) A172 Exp. 1a, MW = 57
9) A172 Exp. 1b, MW = 58
10) A172 Exp. 1c, MW = 57
11) A172 Exp. 2a, MW = 57
12) A172 Exp. 2b, MW = 58
13) A172 Exp. 2c, MW = 58
14) U138MG Exp. 1a, MW = 57
15) U138MG Exp. 1b, MW = 57
16) U138MG Exp. 1c, MW = 57
17) U138MG Exp. 2a, MW = 57
18) U138MG Exp. 2b, MW = 57
19) U138MG Exp. 2c, MW = 52
20) SH-SY5Y Exp. 1c, MW = 58
21) SH-SY5Y Exp. 1a, MW = 52
22) d-SH-SY5Y, Exp. 1a, MW = 58
23) SH-SY5Y Exp. 2b, MW = 58
24) SH-SY5Y Exp. 2a, MW = 57
25) d-SH-SY5Y, Exp. 1b , MW = 57

Exp. – experiment; D2R – dopaminę receptor type 2; d-SH-SY5Y – neuronally differentiated SH-SY5Y cells, not taken for statistical analysis of D2R expression; MW – molecular weight [kDa]

Fig. SII.2. Portrayal of a traditional blot-like image with a lane view of D_3 receptor expression (panel A) and total protein (panel B) of human glioblastoma cell lines (U87MG, A172, U138MG) and human neuroblastoma SH-SY5Y cells probes. Measurments were done on probes from two independent experiments with three repetitions in each (a, b, c).

(A)

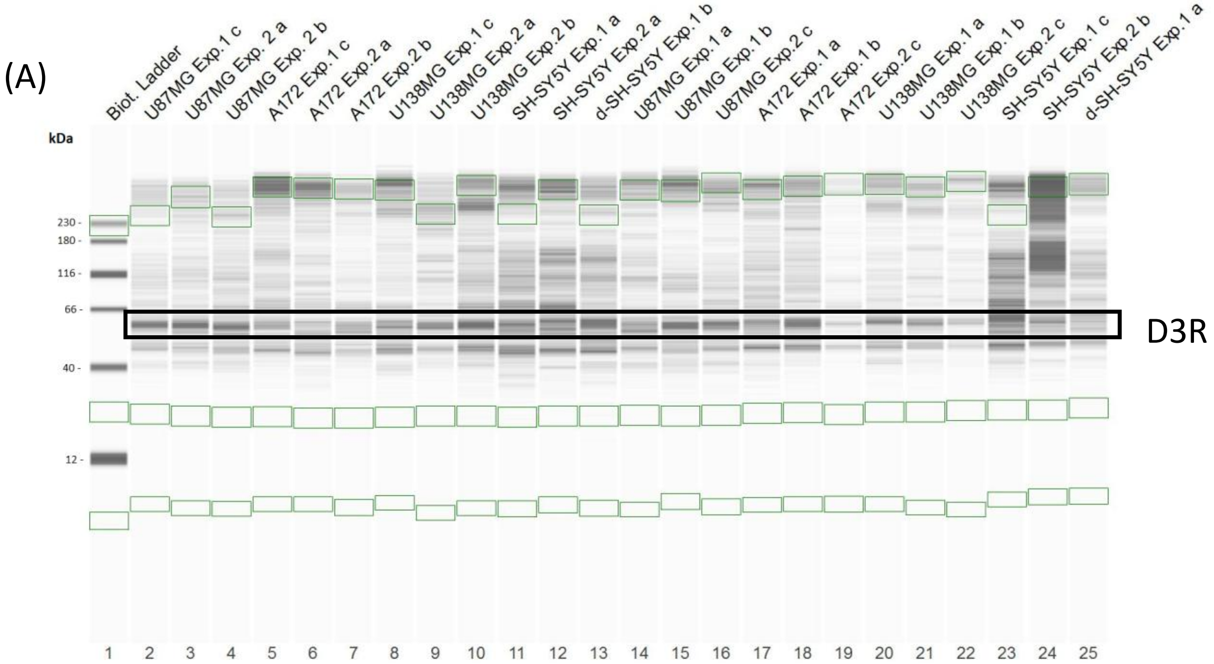

(B)

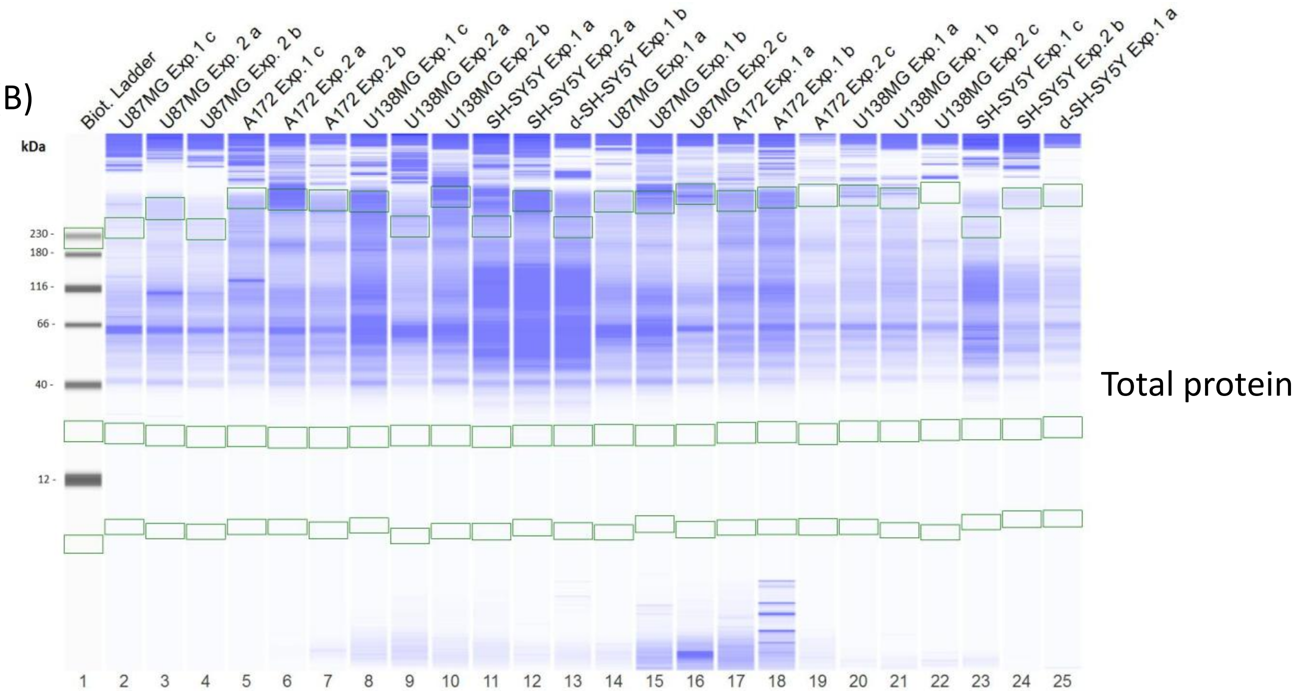

Probes description and MW of D_3R from electropherogram for each probe (53-61 kDa) - 25 probes microcapillary system

1) Biot. Ladder
2) U87MG Exp. 1c, MW = 58
3) U87MG Exp. 2a, MW =55
4) U87MG Exp. 2b, MW = 58
5) A172 Exp. 1c, MW = 53
6) A172p. 2a, MW = 55
7) A172p. 2b, MW = 54
8) U138MG Exp. 1c, MW = 53
9) U138MG Exp. 2a, MW = 57
10) U138MG Exp. 2b, MW = 53
11) SH-SY5Y Exp. 1a, MW = 59
12) SH-SY5Y Exp. 2a, MW = 55
13) d-SH-SY5Y Exp. 1b, MW = 59
14) U87MG Exp. 1a, MW = 54
15) U87MG Exp. 1b, MW = 54
16) U87MG Exp. 2c, MW = 53
17) A172 Exp. 1a, MW = 55
18) A172 Exp. 1b, MW = 51
19) A172 Exp. 2c, MW = 53
20) U138MG Exp. 1a, MW = 54
21) U138MG Exp. 1b, MW = 53
22) U138MG Exp. 2c, MW = 52
23) SH-SY5Y Exp. 1c, MW = 60
24) SH-SY5Y Exp. 2b, MW = 61
25) d-SH-SY5Y Exp. 1a, MW = 61

Exp. – experiment; D3R – dopamine receptor type 3; d-SH-SY5Y – neuronally differentiated SH-SY5Y cells, not taken for statistical analysis of D3R expression; MW – molecular weight [kDa]

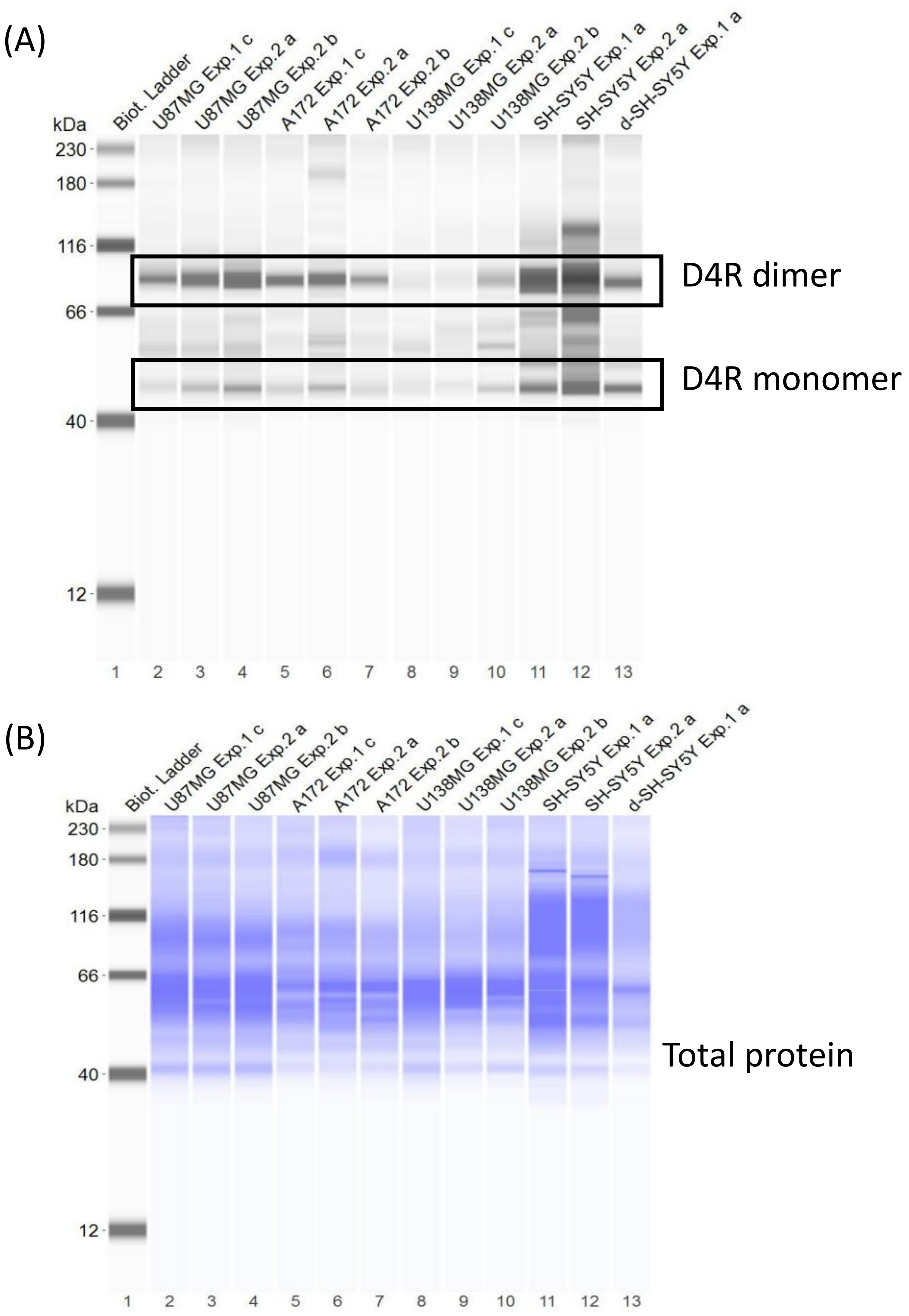

Fig. SII.3. Portrayal of a traditional blot-like image with a lane view of D_4 receptor monomer and dimer expression (panel A), and total protein (panel B) of human glioblastoma cell lines (U87MG, A172, U138MG) and human neuroblastoma SH-SY5Y cells probes. Measurments were done on probes from two independent experiments with three repetitions in each (a, b, c).

Probes description and MW of D_4R monomer (47-49 kDa) and dimer (89-93 kDa) from electropherogram for each probe - 13 probes microcapillary system

1) Biot. Ladder
2) U87MG Exp. 1c, MW = 48; 93
3) U87MG Exp. 2a, MW =49; 91
4) U87MG Exp. 2b, MW = 49, 91
5) A172 Exp. 1c, MW = 48; 90
6) A172 Exp. 2a, MW = 49; 91
7) A172 Exp. 2b, MW = 48; 91
8) U138MG Exp. 1c, MW = 48; 90
9) U138MG Exp. 2a, MW = 48; 92
10) U138MG Exp. 2b, MW = 48; 91
11) SH-SY5Y Exp. 1a, MW = 48; 90
12) SH-SY5Y Exp. 2a, MW = 49; 91
13) d-SH-SY5Y Exp. 1a, MW = 48; 89

Exp. – experiment; D4R – dopamine receptor type 4; d-SH-SY5Y – neuronally differentiated SH-SY5Y cells, not taken for statistical analysis of D4R expression; MW – molecular weight [kDa]

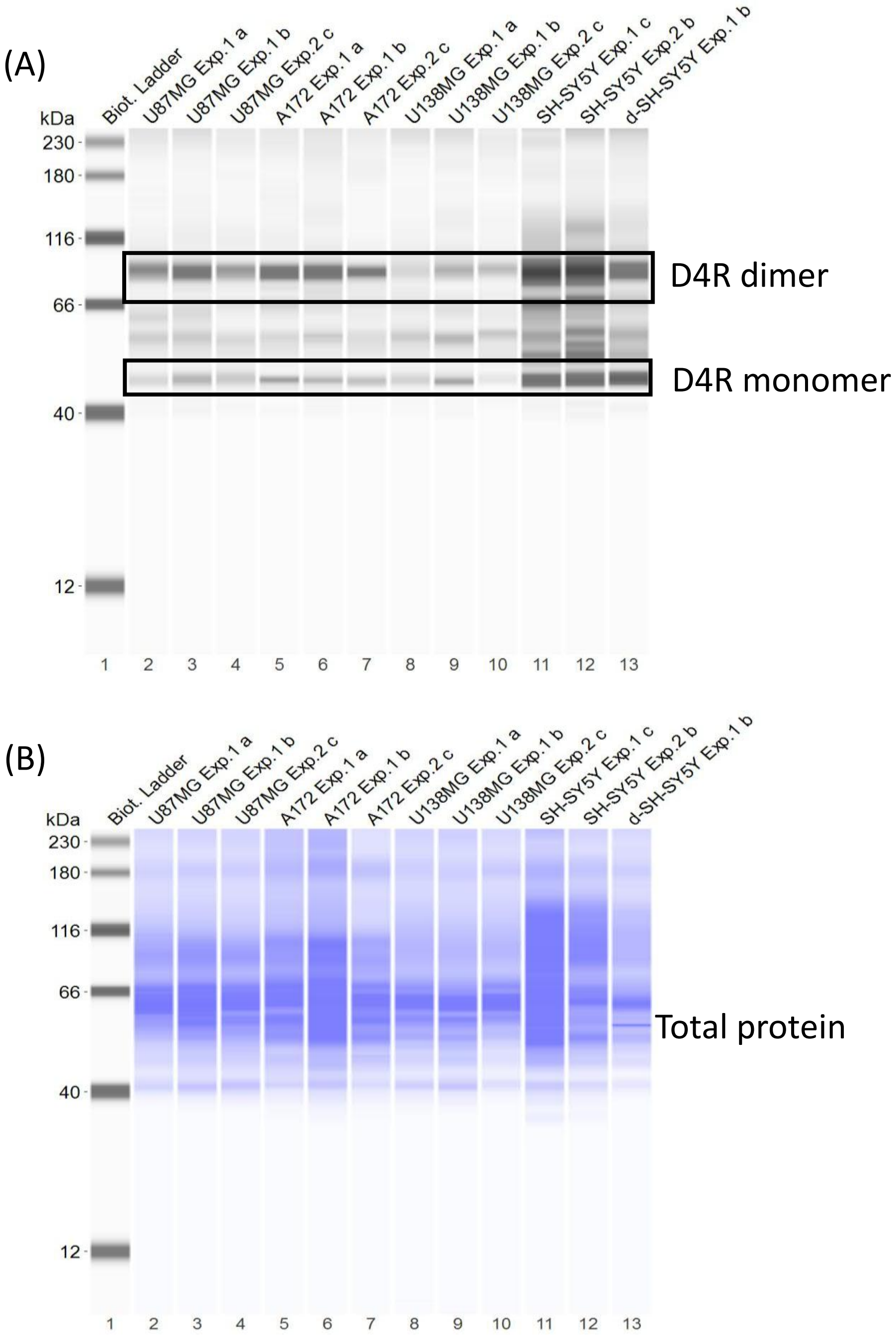

Fig. SII.4. Portrayal of a traditional blot-like image with a lane view of D_4 receptor monomer and dimer expression (panel A), and total protein (panel B) of human glioblastoma cell lines (U87MG, A172, U138MG) and human neuroblastoma SH-SY5Y cells probes. Measurments were done on probes from two independent experiments with three repetitions in each (a, b, c).

Probes description, MW of D4R monomer (47-49 kDa) and dimer (89-93 kDa) from electropherogram for each probe - 13 probes microcapillary system

1) Biot. Ladder,
2) U87MG Exp. 1a, MW = 47; 93
3) U87MG Exp. 1b, MW = 48; 90
4) U87MG Exp. 2c, MW = 48; 91
5) A172 Exp. 1a, MW = 48; 90
6) A172 Exp. 1b, MW = 48;90
7) A172 Exp. 2c, MW = 48; 90
8) U138MG Exp. 1a, MW = 48; 92
9) U138MG Exp. 1b, MW = 48; 96
10) U138MG Exp. 2c, MW = 48; 93
11) SH-SY5Y Exp. 1c, MW = 48; 91
12) SH-SY5Y Exp. 2b, MW = 48; 92
13) d-SH-SY5Y Exp. 1b, MW=48; 92

Exp. – experiment; D4R – dopamine receptor type 4; d-SH-SY5Y – neuronally differentiated SH-SY5Y cells, not taken for statistical analysis of D4R expression; MW – molecular weight [kDa]
